# From Principal Component to Direct Coupling Analysis of Coevolution in Proteins: Low-Eigenvalue Modes are Needed for Structure Prediction

**DOI:** 10.1371/journal.pcbi.1003176

**Published:** 2013-08-22

**Authors:** Simona Cocco, Remi Monasson, Martin Weigt

**Affiliations:** 1 Laboratoire de Physique Statistique de l'Ecole Normale Supérieure - UMR 8550, associé au CNRS et à l'Université Pierre et Marie Curie, Paris, France; 2 Laboratoire de Physique Théorique de l'Ecole Normale Supérieure - UMR 8549, associé au CNRS et à l'Université Pierre et Marie Curie, Paris, France; 3 Université Pierre et Marie Curie, UMR 7238 - Laboratoire de Génomique des Microorganismes, Paris, France; 4 Human Genetics Foundation, Torino, Italy; Linköping University, Sweden

## Abstract

Various approaches have explored the covariation of residues in multiple-sequence alignments of homologous proteins to extract functional and structural information. Among those are principal component analysis (PCA), which identifies the most correlated groups of residues, and direct coupling analysis (DCA), a global inference method based on the maximum entropy principle, which aims at predicting residue-residue contacts. In this paper, inspired by the statistical physics of disordered systems, we introduce the Hopfield-Potts model to naturally interpolate between these two approaches. The Hopfield-Potts model allows us to identify relevant ‘patterns’ of residues from the knowledge of the eigenmodes and eigenvalues of the residue-residue correlation matrix. We show how the computation of such statistical patterns makes it possible to accurately predict residue-residue contacts with a much smaller number of parameters than DCA. This dimensional reduction allows us to avoid overfitting and to extract contact information from multiple-sequence alignments of reduced size. In addition, we show that low-eigenvalue correlation modes, discarded by PCA, are important to recover structural information: the corresponding patterns are highly localized, that is, they are concentrated in few sites, which we find to be in close contact in the three-dimensional protein fold.

## Author Summary

Extracting functional and structural information about protein families from the covariation of residues in multiple sequence alignments is an important challenge in computational biology. Here we propose a statistical-physics inspired framework to analyze those covariations, which naturally unifies existing methods in the literature. Our approach allows us to identify statistically relevant ‘patterns’ of residues, specific to a protein family. We show that many patterns correspond to a small number of sites on the protein sequence, in close contact on the 3D fold. Hence, those patterns allow us to make accurate predictions about the contact map from sequence data only. Further more, we show that the dimensional reduction, which is achieved by considering only the statistically most significant patterns, avoids overfitting in small sequence alignments, and improves our capacity of extracting residue contacts in this case.

## Introduction

Thanks to the constant progresses in DNA sequencing techniques, by now more than 4,400 full genomes are sequenced [1], resulting in more than 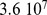 known protein sequences [2], which are classified into more than 14,000 protein domain families [3], many of them containing in the range of 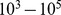 homologous (*i.e.* evolutionarily related) amino-acid sequences. These huge numbers are contrasted by only about 92,000 experimentally resolved X-ray or NMR structures [4], many of them describing the same proteins. It is therefore tempting to use sequence data alone to extract information about the functional and the structural constraints acting on the evolution of those proteins. Analysis of single-residue conservation offers a first hint about those constraints: Highly conserved positions (easily detectable in multiple sequence alignments corresponding to one protein family) identify residues whose mutations are likely to disrupt the protein function, *e.g.* by the loss of its enzymatic properties. However, not all constraints result in strong single-site conservation. As is well-known, compensatory mutations can happen and preserve the integrity of a protein even if single site mutations have deleterious effects [5], [6]. A natural idea is therefore to analyze covariations between residues, that is, whether their variations across sequences are correlated or not [7]. In this context, one introduces a matrix 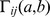 of residue-residue correlations expressing how much the presence of amino-acid ‘
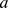
’ in position ‘
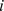
’ on the protein is correlated across the sequence data with the presence of another amino-acid ‘
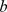
’ in another position ‘
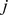
’. Extracting information from this matrix has been the subject of numerous studies over the past two decades, see *e.g.* [5], [6], [8]–[21] and [7] for a recent up-to-date review of the field. In difference to these correlation-based approaches, Yeang *et al.* [22], proposed a simple evolutionary model which measures coevolution in terms of deviation from independent-site evolution. However, a full dynamical model for residue coevolution is still outstanding.

The direct use of correlations for discovering structural constraints such as residue-residue contacts in a protein fold has, unfortunately, remained of limited accuracy [5], [6], [9], [11], [13], [16]. More sophisticated approaches to exploit the information included in 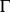 are based on a *Maximum Entropy* (MaxEnt) [23], [24] modeling. The underlying idea is to look for the simplest statistical model of protein sequences capable of reproducing empirically observed correlations. MaxEnt has been used to analyze many types of biological data, ranging from multi-electrode recording of neural activities [25], [26], gene concentrations in genetic networks [27], bird flocking [28] etc. MaxEnt to model covariation in protein sequences was first proposed in a purely theoretical setting by Lapedes *et al.* [29], and applied to protein sequences in an unpublished preprint by Lapedes *et al.* [12]. It was used – even if not explicitly stated – by Ranganathan and coworkers to generate random protein sequences through Monte Carlo simulations, as a part of an approach called Statistical Coupling Analysis (SCA) [15]. Remarkably, many of those artificial proteins folded into a native-like state, demonstrating that MaxEnt modeling was able to statistically capture essential features of the protein family. Recently, one of us proposed, in a series of collaborations, two analytical approaches based on mean-field type approximations of statistical physics, called *Direct Coupling Analysis* (DCA), to efficiently compute and exploit this MaxEnt distribution ([17] uses message passing [19], a computationally more efficient naive mean-field approximation), related approaches developed partially in parallel are [18], [20], [21]. Informally speaking, DCA allows for disentangling direct contributions to correlations (resulting from native contacts) from indirect contributions (mediated through chains of native contacts). Hence, DCA offers a much more accurate image of the contact map than 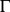 itself. The full potential of maximum-entropy modeling for accurate structural prediction was first recognized in [30] (quaternary structure prediction) and in [31] (tertiary structure prediction), and further applied by [32]–[38]. It became obvious that the extracted information is sufficient to predict folds of relatively long proteins and transmembrane domains. In [36] it was used to rationally design mutagenesis experiments to repair a non-functional hybrid protein, and thus to confirm the predicted structure.

Despite its success, MaxEnt modeling raises several concerns. The number of ‘direct coupling’ parameters necessary to define the MaxEnt model over the set of protein sequences, is of the order of 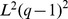. Here, 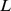 is the protein length, and 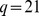 is the number of amino acids (including the gap). So, for realistic protein lengths of 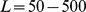, we end up with 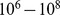 parameters, which have to be inferred from alignments of 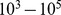 proteins. Overfitting the sequence data is therefore a major risk.

Another mathematically simpler way to extract information from the correlation matrix 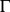 is Principal Component Analysis (PCA) [39]. PCA looks for the eigenmodes of 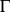 associated to the largest eigenvalues. These modes are the ones contributing most to the covariation in the protein family. Combined with clustering approaches, PCA was applied to identify functional residues in [8]. More recently PCA was applied to the SCA correlation matrix, a variant of the matrix 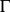 expressing correlations between sites only (and not explicitly the amino-acids they carry) and allowed for identifying groups of correlated (coevolving) residues – termed sectors – each controlling a specific function [40]. A fundamental issue with PCA is the determination of the number of relevant eigenmodes. This is usually done by comparing the spectrum of 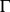 with a null model, the Marcenko-Pastur (MP) distribution, describing the spectral properties of the sample covariance matrix of a set of independent variables [41]. Eigenvalues larger than the top edge of the MP distribution cannot be explained from sampling noise and are selected, while lower eigenvalues – inside the bulk of the MP spectrum, or even lower – are rejected.

In this article we show that there exists a deep connection between DCA and PCA. To do so we consider the Hopfield-Potts model, an extension of the Hopfield model introduced three decades ago in computational neuroscience [42], to the case of variables taking 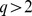 values. The Hopfield-Potts model is based on the concept of patterns, that is, of special directions in sequence space. These patterns show some similarities with sequence motifs or position-specific scoring matrices, but instead of encoding independent-site amino-acid preferences, they include statistical couplings between sequence positions. Some of these patterns are ‘attractive’, defining ‘ideal’ sequence motifs which real sequences in the protein family try to mimic. In distinction to the original Hopfield model [42], we also find ‘repulsive’ patterns, which define regions in the sequence space deprived of real sequences. The statistical mechanics of the inverse Hopfield model, studied in [43] for the 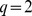 case and extended here to the generic 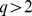 Potts case, shows that it naturally interpolates between PCA and DCA, and allows us to study the statistical issues raised by those approaches exposed above. We show that, in contradistinction with PCA, low eigenvalues and eigenmodes are important to recover structural information about the proteins, and should not be discarded. In addition, we propose a maximum likelihood criterion for pattern selection, not based on the comparison with the MP spectrum. We study the nature of the statistically most significant eigenmodes, and show that they exhibit remarkable features in term of localization: most repulsive patterns are strongly localized on a few sites, generally found to be in close contact on the three-dimensional structure of the proteins. As for DCA, we show that the dimensionality of the MaxEnt model can be very efficiently reduced with essentially no loss of predictive power for the contact map in the case of large multiple-sequence alignments, and with an improved accuracy in the case of small alignments containing too few sequences for standard mean-field DCA to work. These conclusions are established both from theoretical arguments, and from the direct application of the Hopfield-Potts model to a number of sample protein families.

### A short reminder of covariation analysis

Data are given in form of a *multiple sequence alignment* (MSA), in which each row contains the amino-acid sequence of one protein, and each column one residue position in these proteins, which is aligned based on amino-acid similarity. We denote the MSA by 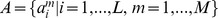 with index 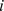 running over the 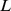 columns of the alignment (residue positions/sites), and 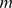 over the 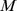 sequences, which constitute the rows of the MSA. The amino-acids 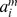 are assumed to be represented by natural numbers 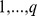 with 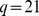, where we include the 20 standard amino acids and the alignment gap ‘-’.

In our approach, we do not use the data directly, but we summarize them by the amino-acid occupancies in single columns and pairs of columns of the MSA (cf. Methods for data preprocessing),
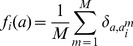
(1)

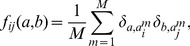
(2)with 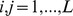 and 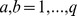. The Kronecker symbol 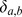 equals one for 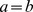, and zero else. Since frequencies sum up to one, we can discard one amino-acid value (*e.g.* 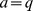) for each position without losing any information about the sequence statistics. We define the empirical covariance matrix through
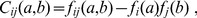
(3)with the position index 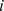 running from 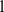 to 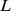, and the amino-acid index from 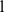 to 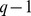. The covariance matrix 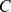 can therefore be interpreted as a square matrix with 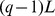 rows and columns. We will adopt this interpretation throughout the paper, since the methods proposed become easier in terms of the linear algebra of this matrix.

#### Maximum entropy modeling and direct couplings

Non-zero covariance between two sites does not necessarily imply the sites to directly interact for functional or structural purposes [13]. The reason is the following [17]: When 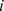 interacts with 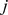, and 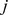 interacts with 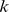, also 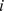 and 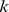 will show correlations even it they do not interact. It is thus important to distinguish between *direct* and *indirect* correlations, and to infer *networks of direct couplings*, which generate the empirically observed covariances. This can be done by constructing a (protein-family specific) statistical model 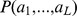, which describes the probability of observing a particular amino-acid sequence 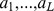. Due to the limited amount of available data, we require this model to reproduce empirical frequency counts for single MSA columns and column pairs,
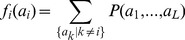
(4)

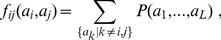
(5)
*i.e.* marginal distributions of 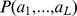 are required to coincide with the empirical counts up to the level of position pairs. Beyond this coherence, we aim at the *least constrained* statistical description. The *maximum-entropy principle* [23], [24] stipulates that 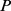 is found by maximizing the entropy
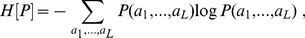
(6)subject to the constraints Eqs. (4) and (5). We readily find the analytical form
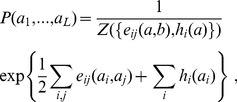
(7)where 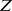 is a normalization constant. The MaxEnt model thus takes the form of a (generalized) 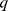-states Potts model, a celebrated model in statistical physics [44], or a Markov random field in a more mathematical language. The parameters 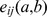 are the direct couplings between MSA columns, and the 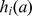 represent the local fields (position-weight matrices) acting on single sites. Their values have to be determined such that Eqs. (4) and (5) are satisfied. Note that, without the coupling terms 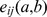, the model would reduce to a standard position-specific scoring matrix. It would describe independent sites, and thus it would be intrinsically unable to capture residue covariation.

From a computational point of view, however, it is not possible to solve Eqs. (4) and (5) exactly. The reason is that the calculations of 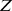 and of the marginals require summations over all 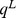 possible amino-acid sequences of length 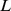. With 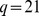 and typical protein lengths of 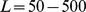, the numbers of configurations are enormous, of the order of 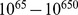. The way out is an approximate determination of the model parameters. The computationally most efficient way found so far is an approximation, called mean field in statistical physics, leading to the approach known as *direct coupling analysis* [19]. Within this mean-field approximation, the values for the direct couplings are simply equal to
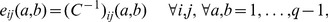
(8)and 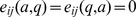 for all 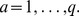 Note that the couplings can be approximated with this formula in a time of the order of 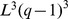, instead of the exponential time complexity, 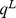, of the exact calculation. On a single desktop PC, this can be achieved in a few seconds to minutes, depending on the length 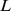 of the protein sequences.

The problem can be formulated equivalently in terms of maximum-likelihood (ML) inference. Assuming 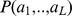 to be a pairwise model of the form of Eq. (7), we aim at maximizing the log-likelihood
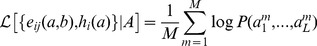
(9)of the model parameters 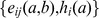 given the MSA 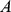. This maximization implies that Eqs. (4) and (5) hold. In the rest of the paper, we will adopt the point of view of ML inference, cf. the details given in Methods. Note that, without restrictions on the couplings 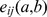 ML and MaxEnt inference are equivalent, but under the specific form for 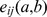 assumed in the Hopfield-Potts model, this equivalence will break down. More precisely, the ML model will fit Eqs. (4) and (5) only approximately

Once the direct couplings 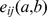 have been calculated, they can be used to make predictions about the contacts between residues, details can be found in the Methods Section. In [19], it was shown that the predictions for the residue-residue contacts in proteins are very accurate. In other words, DCA allows to find a very good estimate of a partial contact map from sequence data only. Subsequent works have shown that this contact map can be completed by embedding it into three dimensions [31], [33].

#### Pearson correlation matrix and principal component analysis

Another way to extract information about groups of correlated residues is the following. From the covariance matrix 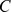 given in Eq. (3), we construct the Pearson correlation matrix 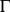 through the relationship
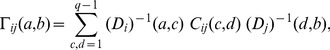
(10)where the matrices 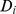 are the square roots of the single-site correlation matrices, *i.e.*

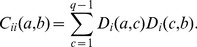
(11)This particular form of the Pearson correlation matrix 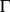 in Eq. (10) results from the fact that we have projected the 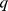-dimensional space defined by the amino-acids 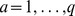 onto the subspace spanned by the first 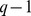 dimensions. Alternative projections lead to modified but equivalent expressions of the Pearson matrix, cf. Text S1 (Sec. S1.3). Informally speaking, the correlation 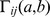 is a measure of comparison of the empirical covariance 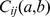 with the single-site fluctuations taken independently. Hence, 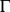 is normalized and coincides with the 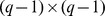 identity matrix on each site: 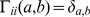.

We further introduce the eigenvalues and eigenvectors (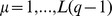)
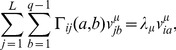
(12)where the eigenvalues are ordered in decreasing order 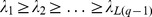. The eigenvectors are chosen to form an ortho-normal basis,
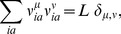
(13)for all 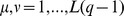. Principal component analysis consists in a partial eigendecomposition of 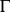, keeping only the eigenmodes contributing most to the correlations, *i.e.* with the largest eigenvalues. All the other eigenvectors are discarded. In this way, the directions of maximum covariation of the residues are identified.

PCA was used by Casari *et al.* [8] in the context of residue covariation to identify functional sites specific to subfamilies of a protein family given by a large MSA. To do so, the authors diagonalized the comparison matrix, whose elements 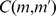 count the number of identical residues for each pair of sequences (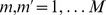). Projection of sequences onto the top eigenvectors of the matrix 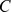 allows to identify groups of subfamily-specific co-conserved residues responsible for subfamily-specific functional properties, called specificity-determining positions (SDP). Up to date, PCA (or the closely related multiple correspondence analysis) is used in one of the most efficient tools, called S3det, to detect SDPs [45]. PCA was also used in an approach introduced by Ranganathan and coworkers [6], [40], called statistical coupling analysis (SCA). In this approach a modified residue covariance matrix, 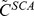, is introduced :
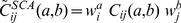
(14)where the weights 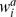 favor positions 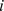 and residues 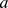 of high conservation. Amino-acid indices are contracted to define the effective covariance matrix,
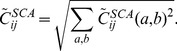
(15)The entries of 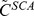 depend on the residue positions 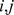 only. In a variant of SCA the amino-acid information is directly contracted at the level of the sequence data. A binary variable is associated to each site: it is equal to one in sequences carrying the consensus amino-acid, to zero otherwise [40]. Principal component analysis can then be applied to the 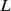-dimensional 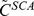 matrix, and used to define so-called sectors, *i.e.* clusters of evolutionarily correlated sites.

## Results

To bridge these two approaches – DCA and PCA – we introduce the Hopfield-Potts model for the maximum likelihood modeling of the sequence distribution, given the residue frequencies 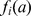 and their pairwise correlations 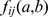. From a mathematical point of view, the model corresponds to a specific class of Potts models, in which the coupling matrix 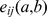 is of low rank 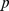 compared to 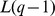. It therefore offers a natural way to reduce the number of parameters far below what is required in the mean-field approximation of [19]. In addition, the solution of the Hopfield-Potts inverse problem, *i.e.* the determination of the low rank coupling matrix 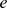, allows us to establish a direct connection with the spectral properties of the Pearson correlation matrix 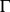 and thus with PCA.

Here, we first give an overview over the most important theoretical results for Hopfield-Potts model inference, increasing levels of detail about the algorithm and its derivation are provided in Methods and Text S1. Subsequently we discuss in detail the features of the Hopfield-Potts patterns found in three different protein families, and finally assess our capacity to detect residue contacts using sequence information alone in a larger test set of protein families.

### Inference with the Hopfield-Potts model

The main idea of this work is that, though the space of sequences is 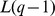
–dimensional, the number of spatial directions being relevant for covariation is much smaller. Such a relevant direction is called *pattern* in the following, and given by a 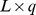 matrix 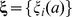, with 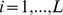 being the site indices, and 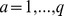 the amino acids. The *log-score* of a sequence 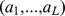 for one pattern 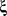 is defined as
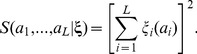
(16)This expression bears a strong similarity with, but also a crucial difference to a position-specific scoring matrix (PSSM): As in a PSSM, the log-score depends on a sum over position and amino-acid specific contributions, but its non-linearity (the square in Eq. (16)) introduces residue-residue couplings, and thus is essential to take covariation into account.

In the Hopfield-Potts model, the probability of an amino-acid sequence 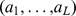 depends on the combined log-scores along a number 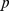 of patterns through
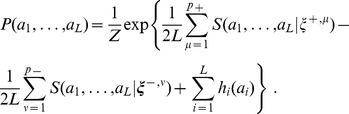
(17)Patterns denoted with a 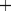-superscript, 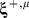 with 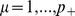, are said to be *attractive*, while the patterns labeled with a 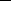-superscript, 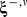 for 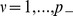, are called *repulsive*. For the probability 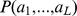 to be large, the log-scores 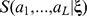 for attractive patterns must be large, whereas the log-scores for repulsive patterns must be small (close to zero). As we will see in the following, the inclusion of such repulsive patterns is important: Compared to the mixed model (17), a model with only attractive patterns achieves a much smaller likelihood (at each given total number of parameters) and a strongly reduced predictivity of residue-residue contacts.

It is easy to see that Eq. (17) corresponds to a specific choice of the couplings 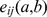 in Eq. (7), namely
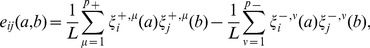
(18)where, without loss of generality, the 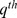 component of the patterns is set to zero, 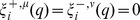, for compatibility with the mean-field approach exposed above. Note that the coupling matrix, for linearly independent patterns, has rank 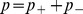, and is defined from 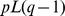 pattern components only, instead of 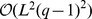 parameters for the most general case of coupling matrices 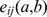. When 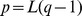, *i.e.* when all the patterns are taken into account, the coupling matrix 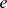 has full rank, and the Hopfield-Potts model is identical to the Potts model used to infer the couplings in DCA in [19]. All results of mean-field DCA are thus recovered in this limiting case.

The patterns are to be determined by ML inference, cf. Methods and Text S1 for details. In mean-field approximation, they can be expressed in terms of the eigenvalues and eigenvectors of the Pearson correlation matrix 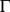, which were defined in Eq. (12). We find that attractive patterns correspond to the 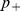 largest eigenvalues (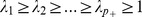),
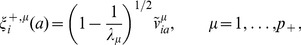
(19)and repulsive patterns to the 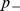 smallest eigenvalues (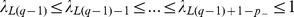),
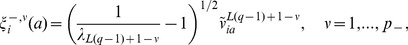
(20)where, for all 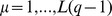,
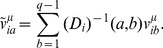
(21)The prefactor 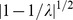 vanishes for 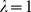. It is not surprising that 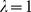 plays a special role, as it coincides with the mean of the eigenvalues:
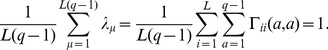
(22)In the absence of any covariation between the residues 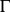 becomes the identity matrix, and all eigenvalues are unity. Hence all patterns vanish, and so does the coupling matrix (18). The Potts model (17) depends only on the local bias parameters 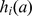, and it reduces to a PSSM describing independent sites.

**Figure 1:**
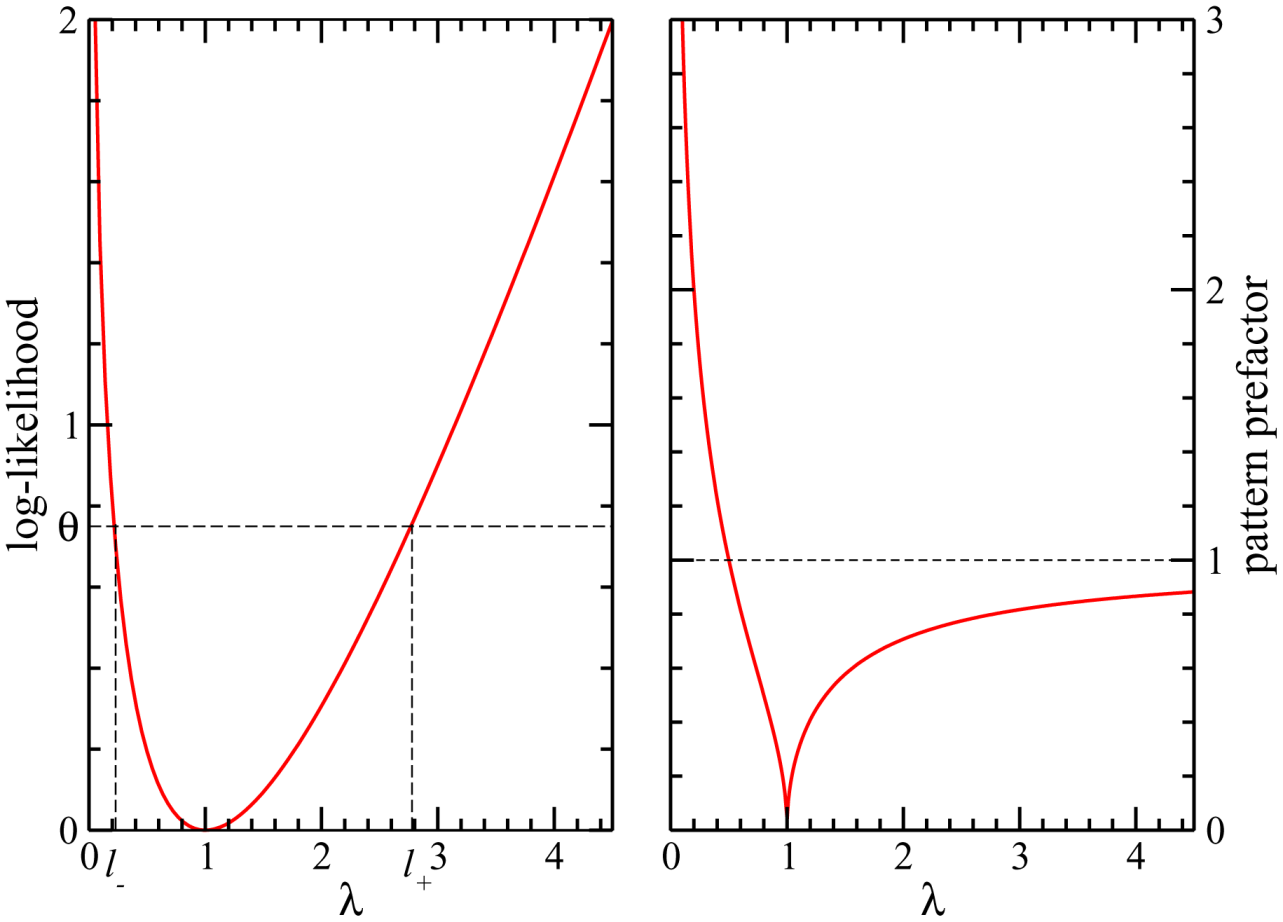
Pattern selection by maximum likelihood and pattern prefactors. *(Left panel)* Contribution of patterns to the log-likelihood (full red line) as a function of the corresponding eigenvalues 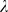 of the Pearson correlation matrix 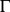. To select 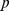 patterns, a log-likelihood threshold 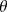 (dashed black line) has to be chosen such that there are exactly 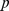 patterns with 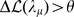. This corresponds to eigenvalues in the left and right tail of the spectrum of 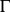. *(right panel)* Pattern prefactors 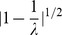 (full red line) as a function of the eigenvalue 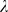. Patterns corresponding to 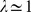 have essentially vanishing prefactors; patterns associated to large 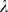 (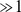) have prefactors smaller than 1 (dashed black line), while patterns corresponding to small 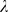 (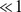) have unbounded prefactors.

The eigenvectors of the correlation matrix with large eigenvalues 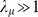 contribute most to the covariation observed in the MSA (i.e. to the matrix 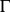), but they do not contribute most to the coupling matrix 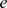. In the expression (18) for this matrix, each pattern carries a prefactor 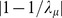: Whereas this prefactor remains smaller than one for attractive patterns (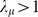), it can become very large for repulsive patterns (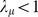), see Fig. 1 (right panel). Thus, the contribution of a repulsive pattern to the 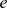 matrix may be much larger than the contribution of any attractive pattern.

Eqs. (19) and (20) *a priori* define 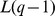 different patterns, therefore we need a rule for selecting the 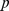 ‘best’, i.e. most likely patterns. We show in Methods that the contribution of a pattern to the model's log-likelihood 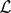 defined in Eq. (9) is a function of the associated eigenvalue 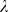 only,
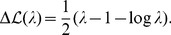
(23)As is shown in Fig. 1 (left panel), large contributions arrive from both the largest and the smallest eigenvalues, whereas eigenvalues close to unity contribute little. According to ML inference, we have to select the 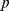 eigenvalues with largest contributions. To this end, we define a threshold value 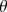 such that there are exactly 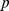 patterns with larger contributions 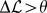 to the log-likelihood; the 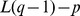 patterns with smaller 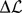 are omitted in the expression for the couplings Eq. (18). In accordance with Fig. 1, we determine thus the two positive real roots 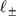 (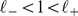) of the equation
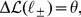
(24)and include all repulsive patterns with 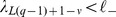, calling their number 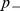, and all attractive patterns with 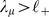, denoting their number by 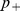. The total number of selected patterns is thus 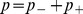.

### Features of the Hopfield-Potts patterns

We have tested the above inference framework in great detail using three protein families, with variable values of protein length 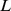 and sequence number 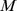:

- The *Kunitz/Bovine pancreatic trypsin inhibitor* domain (PFAM ID PF00014) is a relatively short (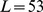) and not very frequent (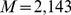) domain, after reweighting the effective number of diverged sequences is 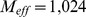 (cf. Eq. (28) in Methods for the definition). Results are compared to the exemplary X-ray crystal structure with PDB ID 5pti [46].
- The bacterial *Response regulator* domain (PF00072) is of medium length (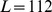) and very frequent (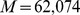). The effective sequence number is 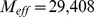. The PDB structure used for verification has ID 1nxw [47].
- The eukaryotic signaling domain *Ras* (PF00071) is the longest (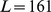) and has an intermediate size MSA (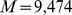), leading to 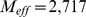. Results are compared to PDB entry 5p21 [48].

In a second step, we have used the 15 protein families studied in [33] to verify that our findings are not specific to the three above families, but generalize to other families. A list of the 15 proteins together with the considered PDB structures is provided in Text S1, Section 4.

To interpret the Hopfield patterns in terms of amino-acid sequences, we first report some empirical observations made for the patterns corresponding to the largest and smallest eigenvalues, *i.e.* to the most likely attractive and repulsive patterns. We concentrate our discussion in the main text on one protein family, the Trypsin inhibitor (PF00014). Analogous properties in the other two protein families are reported in Text S1.

**Figure 2:**
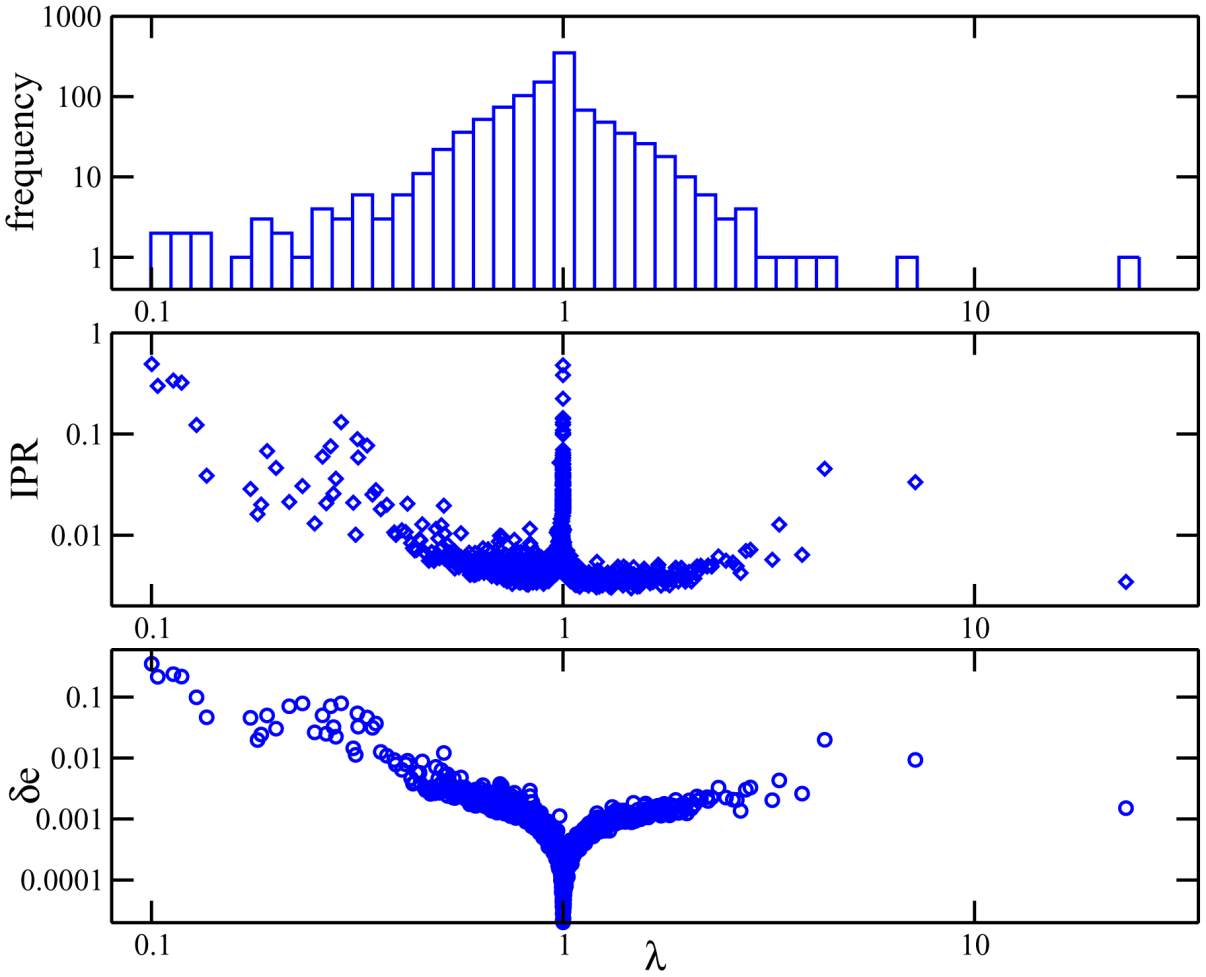
Eigenvalues, localization and contributions to couplings for PF00014. (From top to bottom): *(top panel)* Spectral density as a function of the eigenvalues 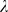, note the existence of few very large eigenvalues, and a pronounced peak in 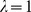. *(middle panel)* Inverse participation ratio of the Hopfield patterns as a function of the corresponding eigenvalue 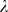. Large IPR characterizes the concentration of a pattern to few positions and amino acids. *(bottom panel)* Typical contribution 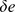 to couplings due to each Hopfield pattern, defined in Eq. (26), as a function of the corresponding eigenvalue 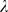. Large contributions are mostly found for small eigenvalues, while patterns corresponding to 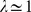 do not contribute to couplings.

The upper panel of Fig. 2 shows the spectral density. It is characterized by a pronounced peak around eigenvalue 1. The smallest eigenvalue is 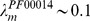, the largest is 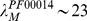. Large eigenvalues are isolated from the bulk of the spectrum, small eigenvalues are not.

To characterize the statistical properties of the patterns we define, inspired by localization theory in condensed matter physics, the inverse participation ratio (IPR) of a pattern 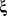 as
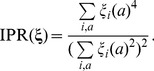
(25)Possible IPR values range from one for perfectly localized patterns (only one single non-zero component) to 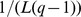 for a completely distributed pattern with uniform entries. IPR is therefore used as a localization measure for the patterns: Its inverse 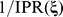 is an estimate of the effective number 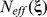 of pairs 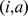, on which the pattern has sizable entries 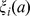. The middle panel of Fig. 2 shows the presence of strong localization for repulsive patterns (small eigenvalues) and for irrelevant patterns (around eigenvalue 1). A much smaller increase in the IPR is also observed for part of the large eigenvalues.

What is the typical contribution 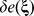 of a pattern 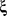 to the couplings? Pattern 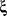 contributes 
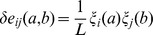
 to each coupling. Many contributions can be small, and others may be larger. An estimate of the magnitude of those relevant contributions can be obtained from the sum of the squared contributions normalized by the effective number 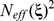 of pairs 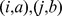 on which the patterns has large entries:
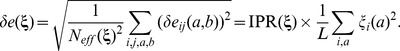
(26)The lower panel of Fig. 2 shows the typical contribution 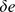 of a pattern as a function of its corresponding eigenvalue. Patterns with eigenvalues close to 1 have very small norms; they essentially do not contribute to the couplings. Highly localized patterns of large norm result in few and large contributions to the couplings (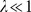). Patterns associated to large eigenvalues 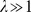 produce many weak contributions to the couplings.

#### Repulsive patterns

**Figure 3:**
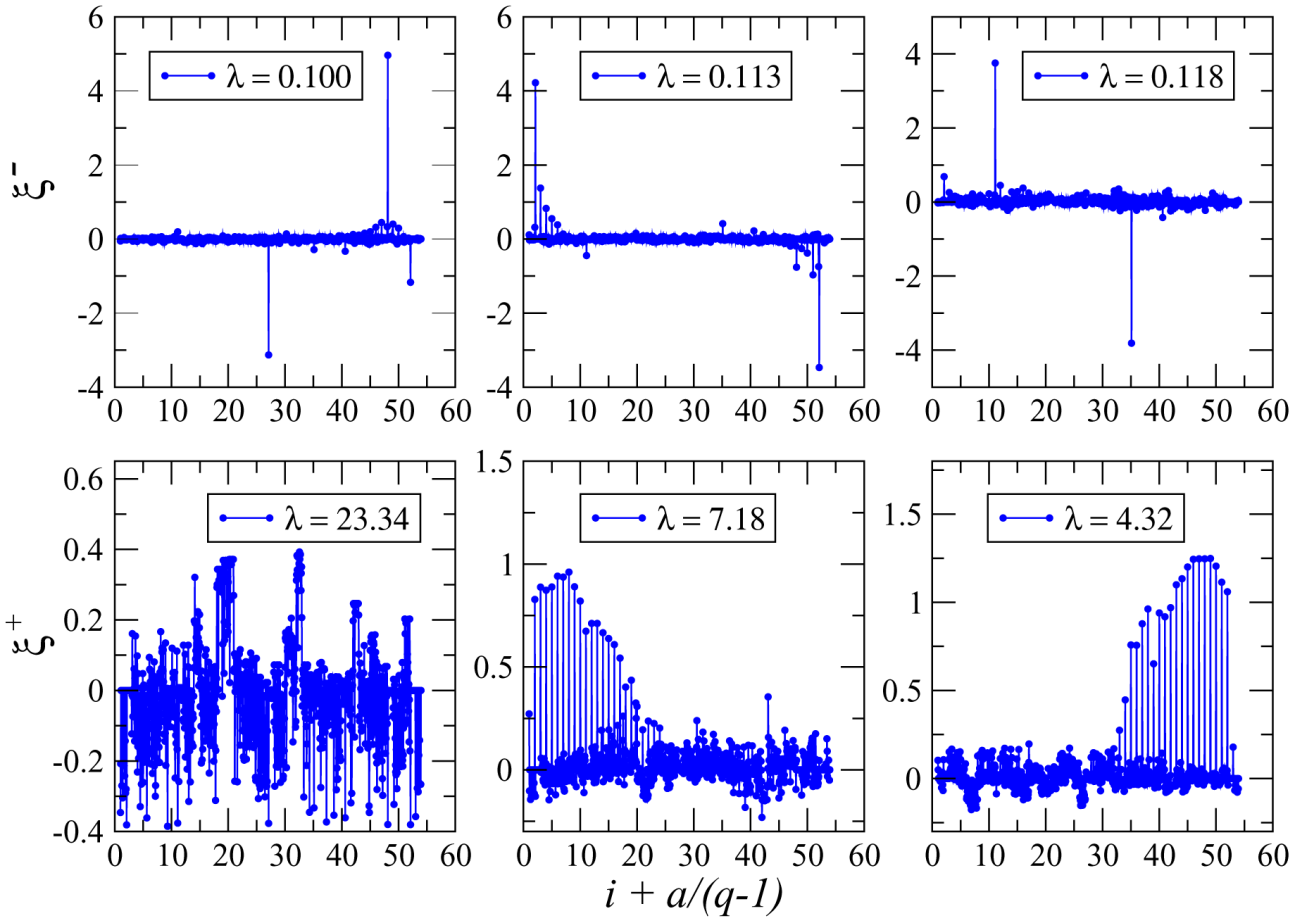
Attractive and repulsive patterns for PF00014. *(Upper panels)* The most localized repulsive patterns (corresponding to the first, third and fourth smallest eigenvalues and inverse participation ratios 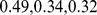 respectively) are strongly concentrated in pairs of positions. *(lower panels)* The most attractive patterns (corresponding to the three largest eigenvalues); the top pattern is extended, with inverse participation ratio 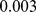, while the second and third patterns,with inverse participation ratios 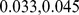 respectively, have essentially non-zero components over the gap symbols only which accumulate on the edges of the sequence. Note the 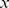-coordinates 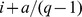; its integer part is the site index, 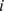, and the fractional part multiplied by 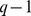 is the residue value, 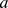.

In the upper row of Fig. 3 we display the three most localized repulsive patterns (smallest, 3rd and 4th smallest eigenvalues) for the trypsin inhibitor protein (PF00014). All three patterns have two very pronounced peaks, corresponding to, say, amino-acid 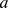 in position 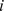 and amino-acid 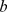 in position 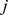, and some smaller minor peaks, resulting in IPR values above 0.3. For each pattern, the two major peaks are of opposite sign: 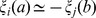. As a consequence, amino-acid sequences carrying amino-acid 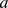 in position 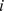, but not 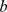 in position 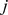 (as well as sequences carrying 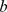 in 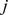 but not 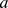 in 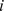) show large log-scores 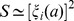, cf. Eq. (16). Their probability in the Hopfield-Potts model, given by (17), will be strongly reduced as compared to the probability of sequences carrying either both amino-acids 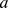 and 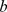 in, respectively, positions 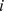 and 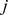, or none of the two (scores 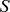 close to zero). Hence, we see that repulsive patterns do define repulsive directions in the sequence space, which tend to be avoided by sequences. A more thorough discussion of the meaning of repulsive patterns will be given in the Discussion Section.

In all three panels of Fig. 3, the two large peaks have highest value for the amino acid cysteine. Actually, for all of them, the pairs of peaks identify disulfide bonds, *i.e.* covariant bonds between two cysteines. They are very important for a protein's stability and therefore highly conserved. The corresponding repulsive patterns forbid amino-acid configurations with a single cysteine in only one out of the two positions. Both residues are co-conserved. Note also that the trypsin inhibitor has only three disulfide bonds, *i.e.* all of them are seen by the most localized repulsive patterns. The second eigenvalues, which has a slightly smaller IPR, is actually found to be a mixture of two of these bonds, *i.e.* it is localized over four positions.

The observation of disulfide bonds is specific to the trypsin inhibitor. In other proteins, also the ones studied in this paper, we find similarly strong localization of the most repulsive patterns, but in different amino acid combinations. As an example, the most localized pattern in the response regulator domain connects a position with an Asp residue (negatively charged), with another position carrying either Lys or Arg (both positively charged), their interaction is thus coherent with electrostatics. In all observed cases, the consequence is a strong statistical coupling of these positions, which are typically found in direct contact.

#### Attractive patterns

**Figure 4:**
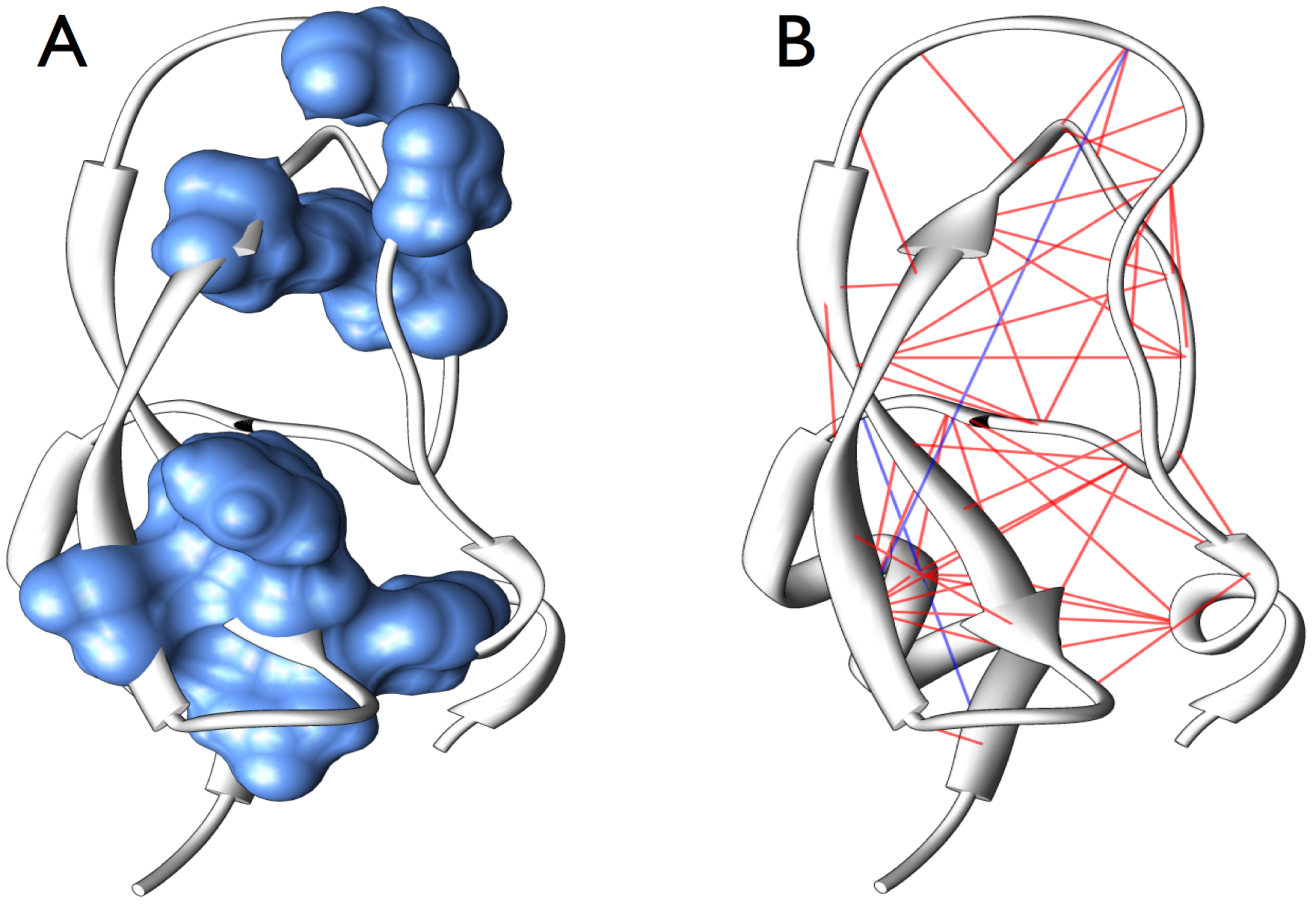
The principal component and predicted contacts visualized on the 3D structure of the trypsin inhibitor protein domain PF00014. *(A)* The 10 positions (residue ID 5,12,14,22,23,30,35,40,51,55) of largest entries in the most attractive Hopfield pattern (largest eigenvalue of 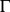, corresponding to the principal component) are shown in blue, they correspond also to very conserved sites. Note that, while they are distant along the protein backbone, they cluster into spatially connected components in the folded protein. *(B)* The 50 residue pairs with strongest couplings (ranked according to the Frobenius norms Eq. (40), with at least 5 positions separation along the backbone, are connected by lines. Only two out of these pairs are not in contact (blue links), all other 48 are thus true-positive contact predictions (red links). Many contacts link pairs of not conserved positions. Note that links are drawn between C-alpha atoms, whereas contacts are defined via minimal all-atom distances, making some red lines to appear rather long even if corresponding to native contacts.

The strongest attractive pattern, *i.e.* the one corresponding to the largest eigenvalue 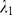, is shown in the leftmost panel of the lower row of Fig. 3. Its IPR is small (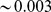), implying that it is extended over most of the protein (a pattern of constant entries would have IPR 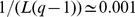). As is shown in Text S1, strongest entries in 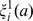 correspond to conserved residues and these, even if they are distributed along the primary sequence, tend to form spatially connected and functionally important regions in the folded protein (*e.g.* a binding pocket), cf. left panel of Fig. 4. Clearly this observation is reminiscent of the protein sectors observed in [40], which are found by PCA applied to the before-mentioned modified covariance matrix. Note, however, that sectors are extracted from more than one principal component.

More characteristic patterns are found for the second and third eigenvalues. As is shown in Fig. 3, they show strong peaks at the extremities of the sequence, which become higher when approaching the first resp. last sequence position. The peaks are, for all relevant positions, concentrated on the gap symbol. These patterns are actually artifacts of the multiple-sequence alignment: Many sequences start or end with a stretch of gaps, which may have one out of at least three reasons: (1) The protein under consideration does not match the full domain definition of PFAM; (2) the local nature of PFAM alignments has initial and final gaps as algorithmic artifacts, a correction would however render the search tools less efficient; (3) in sequence alignment algorithms, the extension of an existing gap is less expensive than opening a new gap. The attractive nature of these two patterns, and the equal sign of the peaks, imply that gaps in equilibrium configurations of the Hopfield-Potts model frequently come in stretches, and not as isolated symbols. The finding that there are two patterns with this characteristic can be traced back to the fact that each sequence has two ends, and these behave independently with respect to alignment gaps.

#### Theoretical results for localization in the limit case of strong conservation

The main features of the empirically observed spectral and localization properties of Fig. 2 can be found back in the limiting case of completely conserved sequences, which is amenable to an exact mathematical treatment. To this end, we consider 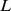 perfectly conserved sites, *i.e.* a MSA made from the repetition of a unique sequence. As is shown in Text S1, Section 2, the corresponding Pearson correlation matrix 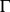 has only three different eigenvalues:

- a large and non-degenerate eigenvalue, 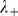, which is a function of 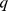 and 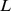 (and of the pseudocount used to treat the data, see Methods), whose corresponding eigenvector is extended;
- a small and 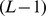-fold degenerate eigenvalue, 
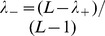
. The corresponding eigenspace is spanned by vectors which are perfectly localized in pairs of sites, with components of opposite signs;
- the eigenvalue 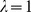, which is 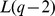-fold degenerate. The eigenspace is spanned by vectors, which are localized over single sites.

For a realistic MSA, *i.e.* without perfect conservation, degeneracies will disappear, but the features found above remain qualitatively correct. In particular, we find in real data a pronounced peak of eigenvalues around 1, corresponding to localized eigenmodes (Fig. 2). In addition, low-eigenvalue modes are found to be strongly localized, and the the order of magnitude of 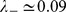 is in good agreement with the smallest eigenvalues, 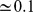, reported for the three analyzed domain families. Finally, the largest eigenmodes are largely extended, as found in the limit case above. Note that the eigenvalues found in the protein spectra, *e.g.* 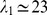 for PF00014, are however smaller than in the limit case, 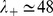, due to only partial conservation in the real MSA.

### Residue-residue contact prediction with the Hopfield-Potts model

The most important feature of DCA is its ability to predict pairs of residues, which are distantly positioned in the sequence, but which form native contacts in the protein's tertiary structure, cf. the right panel of Fig. 4. Here, our contact prediction is based on the sampling-corrected Frobenius norm of the 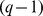
–dimensional statistical coupling matrices 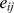, cf. Methods, which in [49] has been shown to outperform the direct-information measure used in [17]. This measure assigns a single scalar value for the strength of the direct coupling between two residue positions.

**Figure 5:**
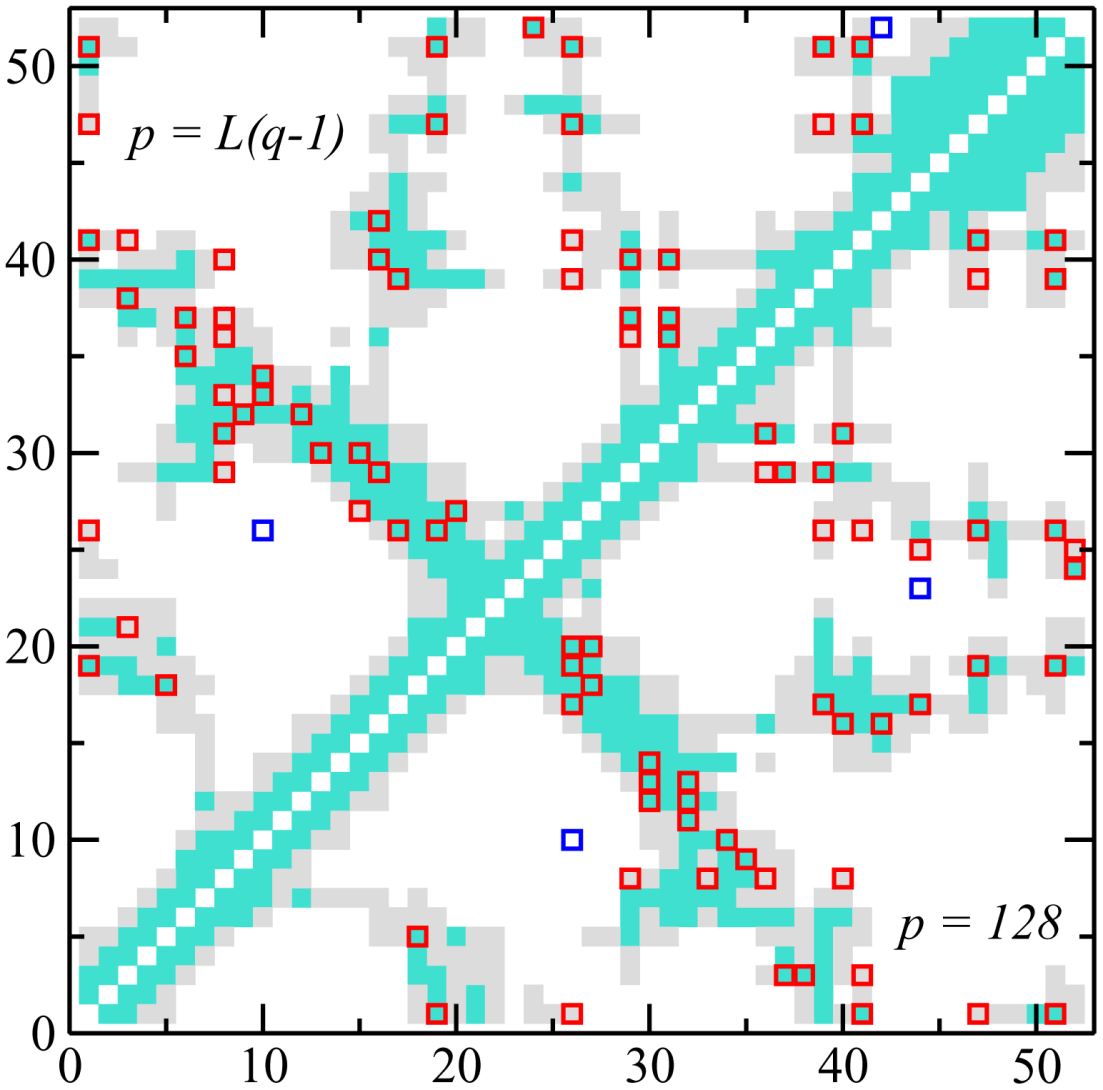
Contact map for the PF00014 family. Filled squares represent the native contact map on the 3D fold (PDB 5pti, with turquoise squares signaling all-atom distances below 5 Å, and grey ones distances between 5 Å and 8 Å). The 50 top predicted contacts with minimal separation of 5 positions along the sequence (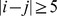) are shown with empty squares: true-positive predictions (distance 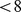
Å) are colored in red, and false-positive predictions in blue. Predictions are made with the Hopfield-Potts model with 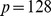 patterns (bottom right corner) and with 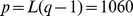 patterns (DCA, top left corner). For both values of 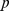 there are 48 true-positive and 2 false-positive predictions.

**Figure 6:**
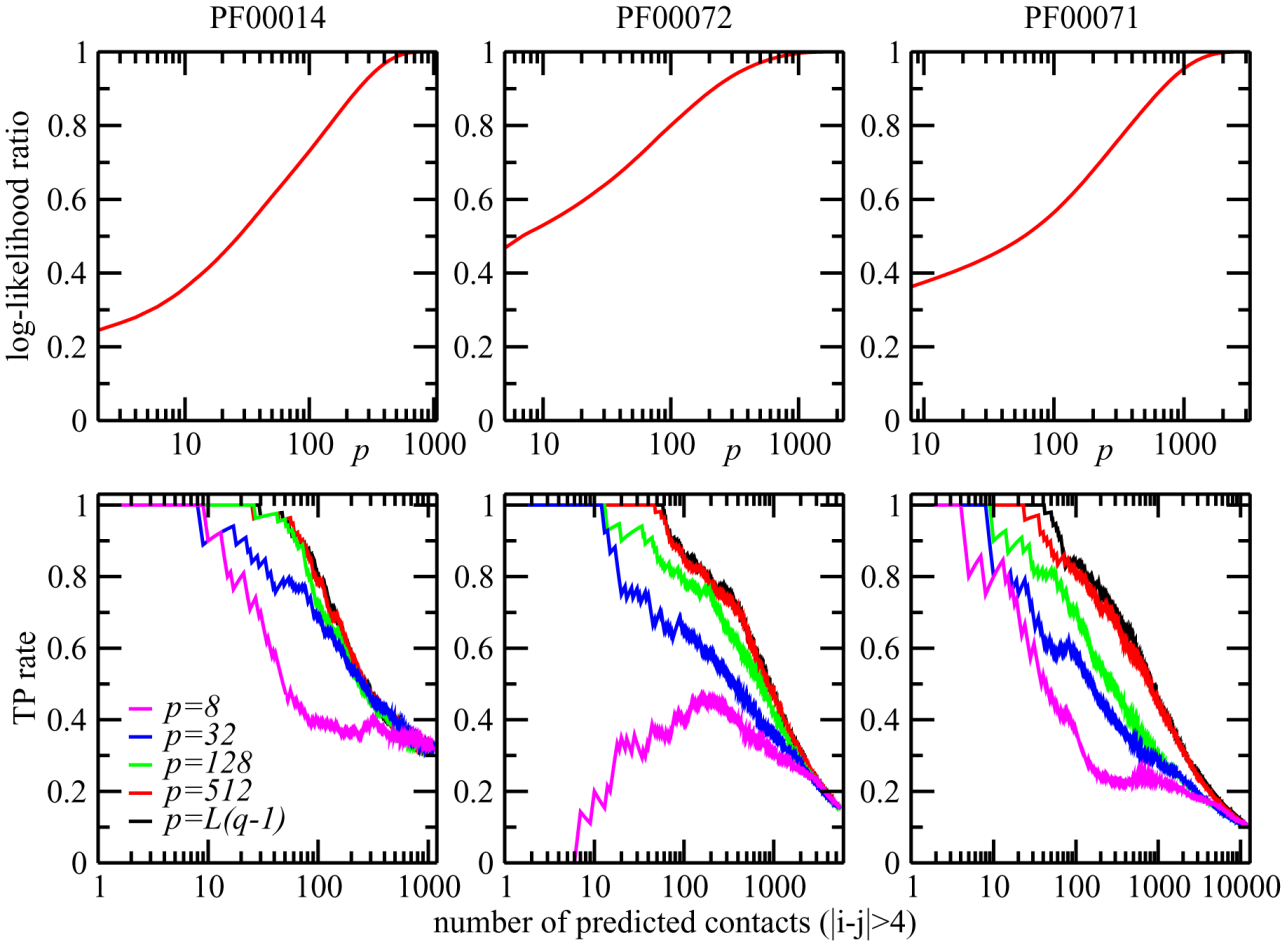
Contact predictions for the three considered protein families. The upper panels show the fraction of the interaction-based contribution to the log-likelihood of the model given the MSA, defined as the ratio of the log-likelihood with 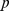 selected patterns over the maximal log-likelihood obtained by including all 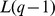 patterns, as a function of the number 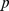 of selected patterns, it reaches one for 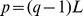 corresponding to the Potts model used in DCA. The lower panels show the TP rates as a function of the predicted residue contacts, for various numbers 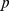 of selected patterns, where selection was done using the maximum-likelihood criterion. 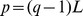 gives the contact predictions obtained by DCA approach. Only non-trivial contacts between sites 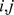 such that 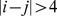 are considered in the calculation of the TP rate.

The contact map predicted from the 50 strongest direct couplings for the PF00014 family is compared to the native contact map in Fig. 5. In accordance with [19], a residue pairs is considered to be a true positive prediction if its minimal atom distance is below 8 Å in the before mentioned exemplary protein crystal structures. This relatively large cutoff was chosen since DCA was found to extract a bimodal signal with pairs in the range below 5 Å (turquoise in Fig. 5) and others with 7–8 Å (grey in Fig. 5); both peaks contain valuable information if compared to typical distances above 20 Å for randomly chosen residue pairs. To include only non-trivial contacts, we require also a minimum separation 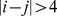 of at least 5 residues along the protein sequence. Remarkably the quality of the predicted contact map with the Hopfield-Potts model with 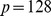 patterns is essentially the same as with DCA, corresponding to 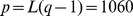 patterns. In both cases predicted contacts spread rather uniformly over the native contact map, and 96% of the predicted contacts are true positives. This result is corroborated by the lower panels of Fig. 6, which show, for various values of the number 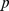 of patterns, the performance in terms of contact predictions for the three families studied here. The plots show the fraction of true-positives (TP), *i.e.* of native distances below 8 Å, in between the 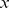 pairs of highest couplings, as a function of 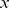 [19].

The three upper panels in Fig. 6 show the ratio between the selected pattern contributions to the log-likelihood, 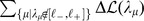, and its maximal value obtained by including all 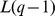 patterns, 
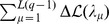
. A large fraction of patterns can be omitted without any substantial loss in log-likelihood, but with a substantially smaller number of parameters. It is worth noting that, in Fig. 6, we do not find any systematic benefit of excluding patterns for the contact prediction, but the predictive power decreases initially only very slowly with decreasing pattern numbers 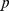. For all three proteins, even with 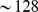 patterns, very good contact predictions can be achieved, which are comparable to the ones with 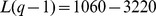 patterns using the full DCA inference scheme of [19]. Almost perfect performance is reached, when the contribution of selected patterns to the log-likelihood is only at 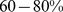 of its maximal value. This could be expected from the fact that patterns corresponding to eigenvalues close to unity hardly contribute to the couplings, cf. lower panel in Fig. 2.

**Figure 7:**
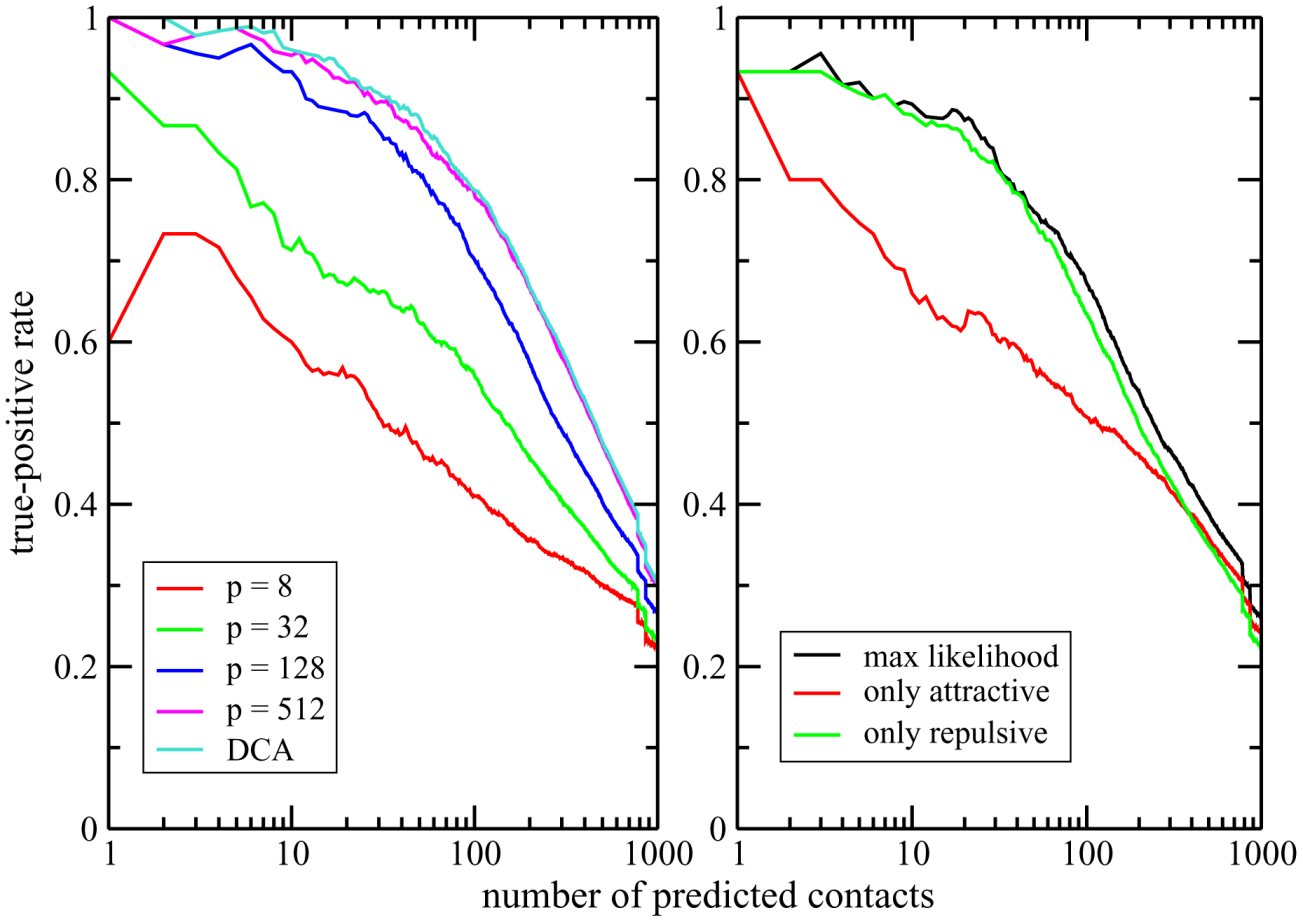
Contact predictions across 15 protein families. *(Left panel)* TP rates for the contact prediction with variable numbers 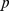 of Hopfield-Potts patterns, averaged over 15 distinct protein families. *(right panel)* TP rates for the contact prediction using only the repulsive (green line) resp. attractive (red line) patterns, which are contained in the 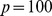 most likely patterns (black line), averaged over 15 protein families. It becomes obvious that the contact prediction remains almost unchanged when only the subset of repulsive patterns is used, whereas it drops substantially by keeping only attractive patterns.

These findings are not restricted to the three test proteins, as is confirmed by the left panel of Fig. 7. In this figure, we average the TP rates for 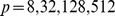 and 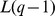 (*i.e.* full mean-field DCA) for the 15 proteins studied in [33], which had been selected for their diversity in protein length and fold type. Further more, the discussion of the localization properties of repulsive patterns is corroborated by the results reported in Fig. 7, right panel. It compares the performance of the Hopfield-Potts model to predict residue-residue contacts, for the three cases where 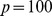 patterns are selected either according to the maximum likelihood criterion (patterns for eigenvalues 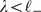 and for 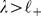), or where only the strongest attractive (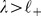) or only the strongest repulsive (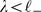) patterns are taken into account. It becomes evident that repulsive patterns provide more accurate contact information, TP rates are almost unchanged between the curve of the 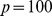 most likely patterns, and the smaller subset of repulsive patterns. On the contrary, TP rates for contact prediction are strongly reduced when considering only attractive patterns, *i.e.* in the case corresponding most closely to PCA. This finding illustrates one of the most significative differences between DCA and PCA: Contact information is provided by the eigenvectors of the Pearson correlation matrix 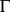 in the lower tail of the spectrum.

As is discussed in the previous section, patterns with the largest contribution to the log-likelihood are dominated by (and localized in) conserved sites. Attractive patterns favor these sites to jointly assume their conserved values, whereas repulsive patterns avoid configurations where, in pairs of co-conserved sites, only one variable assumes its conserved value, but not the other one. However, we have also seen that an accurate contact prediction requires at least 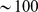 patterns, *i.e.* it goes well beyond the patterns given by strongly conserved sites. In Fig. 4 we show, for the exemplary case of the Trypsin inhibitor, both the 10 sites of highest entry in the most attractive pattern 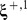 (corresponding to conserved sites), and the first 50 predicted intra-protein contacts using the full mean-field DCA scheme (results for 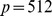 are almost identical). It appears that many of the correctly predicted contacts are not included in the set of the most conserved sites. From a mathematical point of view, this is understandable - only variable sites may show covariation. From a biological point of view, this is very interesting, since it shows that highly variable residue in proteins are not necessarily functionally unimportant in a protein family, but they may undergo strong coevolution with other sites, and thus be very important for the structural stability of the protein, cf. also the Fig. S5 in Text S1 where the degree of conservation [50] is depicted for the highest-ranking DCA predicted contacts. In this figure we show that residues included in predicted contacts are found for all levels of conservation. It has, however, to be mentioned that in the considered MSA, there are no 100% conserved residues, the latter would not show any covariation. A small level of variability is therefore crucial for our approach.

A remark is necessary concerning the right panel of Fig. 4: Whereas conserved sites (which carry also the largest entries of the pattern with maximum eigenvalue) are collected in one or two spatially connected regions in the studied proteins, this is not necessarily true for all proteins. In particular complex domains with multiple functions and/or multiple conformations may show much more involved patterns. It is, however, beyond the scope of this paper to shed light onto the details of the biological interpretation of the principal components of 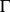.

**Figure 8:**
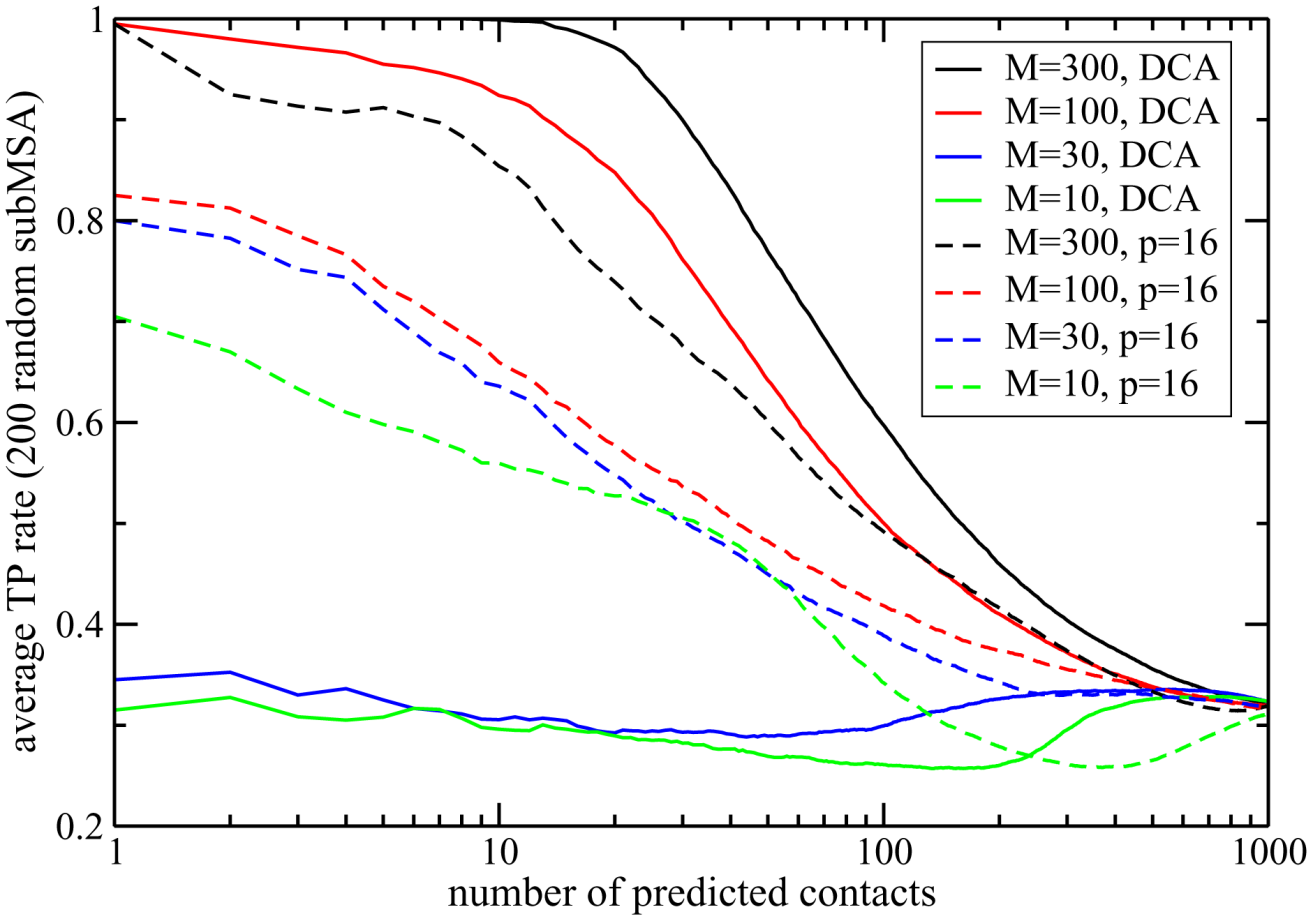
Noise reduction due to pattern selection in reduced data sets. *(Full lines)* TP rates of mean-field DCA for sub-MSAs of family PF00014 with 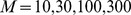 sequences; each curve is averaged over 200 randomly selected sub-alignments. Whereas for 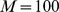 and 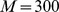 the accuracy of the first predictions is close to one, mean-field DCA does not extract any reasonable signal for 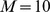 and 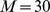. *(dashed lines)* The same sub-MSA are analyzed with the Hopfield-Potts model using 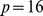 patterns (maximum-likelihood selection). Whereas this selection reduces the accuracy for 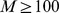, it results in increased TP rates for 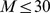. Dimensional reduction by pattern selection has lead to an efficient noise reduction.

In which cases does the dimensional reduction achieved by selecting only a relatively small number of patterns provide an actual advantage over the standard mean-field DCA approach with 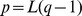 patterns? We have seen that for relatively large MSAs, where DCA gives very accurate results, the approach presented here achieves a strong dimensional reduction almost without loss in predictive power, but it did not improve the contact map prediction, cf. Figs. 5 and 6. However, when we reduce the number of sequences in the MSA, DCA undergoes a strong reduction in accuracy of prediction, see the full lines in Fig. 8 where DCA is applied to sub-alignments of the PF00014 domain family. Repeating the same experiment with a finite number of patterns (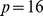 in Fig. 8), the MSA-size dependence is strongly reduced. For very small alignments of only 10–30 sequences, the Hopfield-Potts model is still able to extract contacts with an astonishing TP rate of 70–80%, whereas DCA produces almost random results (TP rate ca. 30%). The success of the Hopfield-Potts approach for small MSA is not specific to the PF00014 domain, and holds for other protein families, see Fig. S15 in Text S1. Hopfield-Potts patterns are therefore an efficient means to reduce overfitting effects found in DCA, and to improve the signal-to-noise ratio.

## Discussion

In this paper we have proposed a method to analyze the correlation matrix of residue occurrences across multiple-sequence alignments of homologous proteins, based on the inverse Hopfield-Potts model. Our approach offers a natural interpolation between the spectral analysis of the correlation matrix, carried out in principal component analysis (PCA), and maximum entropy approaches which aim at reproducing those correlations within a global statistical model (*e.g.* DCA). The inverse Hopfield-Potts model requires to infer “directions” of particular importance in the sequence space, called patterns: The distribution of sequences belonging to a protein family tends to accumulate along attractive patterns (related to eigenmodes of the correlation matrix with large eigenvalues) and to get depleted around repulsive patterns (related to the low-eigenvalue modes). These patterns have some similarity with position-specific scoring matrices frequently used in the statistical modeling of sequences, but in contrast to the independence of different positions in PSSM, Hopfield-Potts patterns account for inter-position couplings, as needed for coevolutionary analysis.

Contrary to principal component analysis, which discards low-eigenvalue modes, we have shown that repulsive patterns are essential to characterize the sequence distribution, and in particular to detect structural properties (residue-residue contacts) of proteins from sequence data. In addition, we have shown how to infer not only the values of the patterns but also their statistical relevance from the sequence data. To do so, we have calculated the contribution of each pattern to the total likelihood of the Hopfield-Potts model given the data, establishing thus a clear criterion for pattern selection. The results of the application of the inverse Hopfield-Potts model to real sequence data confirm that most eigenmodes (with eigenvalues close to unity) can be discarded without affecting considerably the contact prediction (see Fig. 5 and Fig. 6). This makes our approach much less parameter-intensive that the full direct coupling analysis DCA. We have found empirically that it is sufficient to take into account the patterns contributing to 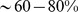 of the log-likelihood to achieve a very good contact map prediction in the case of large multiple-sequence alignments. In the case of reduced MSA size, we found that the dimensional reduction due selecting only the most likely patterns improves the signal-to-noise ratio of the inferred model, and therefore reaches a better contact prediction than mean-field DCA, down to very small numbers of sequences, see Fig. 8 and Fig. S15 in Text S1. Moreover the Hopfield-Potts approach can be very advantageous in terms of computational time. While DCA requires the inversion of the correlation matrix, which takes 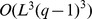 time, computing the 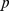 patterns (corresponding to the largest and smallest eigenvalues) can be done in 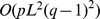 time only. The reduction in computational time can thus be very important for large proteins.

We have also studied the position-specific nature of patterns, taking inspiration from localization theory in condensed matter physics and random matrix theory (Fig. 3 and Figs. S8 and S12 in Text S1). Briefly speaking, a pattern is said to be localized if it is concentrated on a few sites of the sequence, and extended (over the sequence) otherwise. We have found that the principal attractive pattern (corresponding to the largest eigenvalue) is extended, with entries of largest absolute value in the most conserved sites (Figs. S3, S4, S9 & S13 in Text S1). Other strongly attractive patterns can be explained from the presence of extended gaps in the alignment, mostly found at the beginning or at the end of sequences. The other patterns of large likelihood contributions are repulsive, *i.e.* they correspond to small eigenvalues, usually discarded by principal component analysis. Interestingly, these patterns appear to be strongly localized, that is, strongly concentrated in very few positions, which despite their separation along the sequence are found in close contact in the 3D protein structure. To give an example, in the Trypsin inhibitor protein, they are localized in position pairs carrying Cysteine, and being linked by disulfide bonds. Other amino-acid combinations were also found in the other protein families studied here, e.g. patterns connecting residues of opposite electrical charge. Taking into account only a number 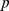 of such repulsive patterns results in a predicted contact map of comparable quality to the one using maximum-likelihood selection, whereas the same number 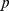 of attractive patterns performs substantially worse (Fig. 7 and Fig. S7 & S11in Text S1). The dimensional reduction of the Hopfield-Potts model compared to the Potts model (used in standard DCA) is thus even more increased as many relevant patterns are localized and contain only a few (substantially) non-zero components. As a consequence the couplings found with the Hopfield-Potts model are sparser than their DCA counterparts (Fig. S6 in Text S1).

It is important to stress that also distinct patterns, whether attractive or repulsive, can have large components on the same sites and residues. A general finding, supported by a theoretical analysis in the Results section, is that the more repulsive patterns are, the stronger they are localized, and the more conserved are the residues supporting them. Highly conserved sites therefore appear both in the most attractive pattern and, when covarying with other residues, in a few localized and repulsive patterns reflecting those covariations. As the number of patterns to be included to reach an accurate contact prediction is a few hundreds for the protein families considered here, the largest components of the weakly repulsive patterns, *i.e.* with the eigenvalues smaller than, but close to the threshold 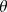, correspond to weakly conserved residues. In consequence many predicted contacts connect low-conservation residues. This statement is apparent from Fig. 4 and Figs. S10 and S14 in Text S1, which compare the sets of conserved sites and the pairs of residues predicted to be in contact by our analysis.

Why are repulsive patterns so successful in identifying contacts, in difference to attractive patterns? To answer this question, consider the simple case of a pattern 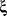 localized in two residues only, say it should prefer the co-occurrence of amino-acid 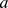 in position 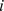, and of amino acid 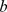 in position 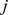. We further assume that the two non-zero components 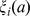 and 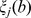 have the same amplitude and differ only by sign, *i.e.* 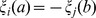. Now we consider a sequence of amino-acids 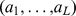 and ask whether it will have a large log-score 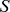 for pattern 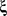, see Eq. (16). The outcome is given in the third column of Table 1. The log-score therefore corresponds to a XOR (exclusive or) between the presence of the two amino-acids 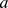 and 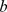 on their respective positions 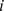 and 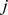 in the sequence. If the pattern were attractive (cf. fourth column), it would favor sequences where exactly one of the two specified amino-acids is present. For a repulsive pattern (cf. fifth column), low log-score sequences are favored, *i.e.* either both 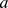 and 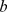 are present in positions 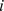 and 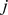, or none of the two.

**Table 1:** Effect of a pattern with two non-zero and opposite components 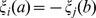.

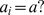	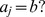	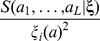	Favored by attractive pattern?	Favored by repulsive pattern?
NO	NO	0	NO	YES
YES	NO	1	YES	NO
NO	YES	1	YES	NO
YES	YES	0	NO	YES

In case we assumed equal sign components, *i.e.* 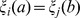, we would have found Table 2. This choice is poor in terms of enforcing covariation in the sequence: An attractive (resp. repulsive) pattern strongly favors (resp. disfavors) the simultaneous presence of amino acids 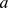 and 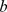 in positions 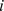 and 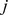, but the likelihood is monotonous in the number of correctly present amino acids.

**Table 2:** Effect of a pattern with two non-zero and equal components 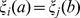.

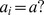	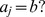	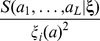	Favored by attractive pattern?	Favored by repulsive pattern?
NO	NO	0	NO	YES
YES	NO	1	NO	YES
NO	YES	1	NO	YES
YES	YES	4	YES	NO

As a conclusion, we find that strong covariation can be efficiently enforced only by a repulsive pattern with opposite components (fifth column in Table 1). The acceptance of the (NO,NO) configuration is desirable, too: It signals the possibility of compensatory mutations, *i.e.* favorable double mutations changing both 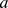 and 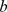 in positions 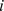 and 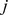 to alternative amino acids. It is easy to generalize the above patterns to patterns having more than one favored amino-acid combination, *e.g.* favored pairings 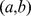 and 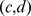 can be enforced by a repulsive pattern with 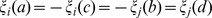. This theoretical argument explains why localized repulsive patterns critically encode for covariation. Remarkably the condition that the few, large components of repulsive patterns should sum up to zero agrees well with our findings in real MSAs, cf Fig. 3 and Figs. S8 and S12 in Text S1. Furthermore, it would be interesting to better understand the relationship between such localized patterns and specificity-determining positions [8], [45]: SDP are co-conserved in subfamilies of the full MSA, but vary from one family to another. The most repulsive patterns are localized in residues, which are strongly conserved throughout the full alignment. We have also used S3det [45] to predict SDPs and to compare them to our 30 highest-scoring contact predictions, and we have not observed any particular signal. It would be interesting to extend the Hopfield-Potts approach to subfamilies and to investigate, if SDPs correspond to repulsive patterns in these subfamilies.

Last but not least, let us emphasize the importance of the prefactor 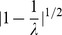 of the pattern, cf. Eqs. (19) and (20), where 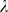 is the eigenvalue corresponding to the pattern. While this factor is at most equal to 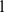 for attractive patterns, it can take arbitrarily large values for repulsive patterns (Fig. 1, right panel). Moreover, repulsive patterns can be highly localized: they strongly contribute to a few couplings 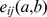, *e.g.* to one coupling between a single pair of positions 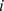 and 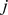 for patterns perfectly localized in two sites only (cf. Fig. 2, lower panel, and Fig. 3). Consequently those contributions are of particular importance in the ranking of couplings, which our contact prediction is based on. On the contrary, attractive patterns, even with sizeable norms, produce many weaker contributions to the couplings (cf. Fig. 2, lower panel), and do not alter their relative rankings a much as repulsive patterns do. This explains why contact prediction based on repulsive patterns only is much more efficient than when based on attractive patterns only (cf. Fig. 7).

Some aspects of the approach presented in this paper deserve further studies, and may actually lead to substantial improvements of our ability to detect residue contacts from statistical sequence analysis. The probably most important question is the capability of our approach to suppress noise in small MSAs, and to extract contact information in cases where mean-field DCA fails. This question is closely related to the determination of an optimal value for the pattern number 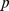 using sequence information alone. Second, the non-independence of sequences in the alignment, *e.g.* due to phylogenetic correlations, should be taken into account in a more accurate way than done currently by sequence reweighting. Third, the precise role of the – heuristically determined – large pseudo-count used to calculate the Pearson correlation matrix should also be elucidated. Fourth, while the use of the Frobenius norm for the coupling 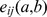 (with the average-product correction, see Methods) has proven to be an efficient criterion for contact prediction, it remains unclear if there exist other contact estimators with better performance. In this context it would also be interesting to find a threshold for these contact scores, which separates a signal-rich from a noise-dominated region. And last but not least, it would be interesting to integrate prior knowledge about proteins, like *e.g.* amino-acid properties or predicted secondary structure, into the purely statistical inference approach presented here.

The MATLAB program necessary for the analysis of the data, the computation of the patterns, and the contact prediction is available as part of the Supporting Information. Users of the program are kindly requested to cite the present work.

## Methods

### Data preprocessing

Following the discussion of [19], we introduce two modifications into the definition Eq. (2) of the frequency counts 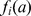 and 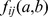:

- *Pseudocount regularization*: Some amino-acid combinations 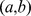 do not exist in column pairs 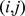, even if 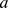 is found in 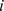, and 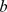 in 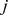. This would formally lead to infinitely large coupling constants, and the covariance matrix 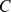 becomes non invertible. This divergence can be avoided by introducing a pseudocount 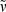, which adds to the occurrence counts of each amino acid in each column of the MSA.
- *Reweighting*: The sampling of biological sequences is far from being identically and independently distributed (i.i.d.), it is biased by the phylogenetic history of the proteins and by the human selection of sequenced species. This bias will introduce global correlations. To reduce this effect, we decrease the statistical weight of sequences having many similar ones in the MSA. More precisely, the weight of each sequence is defined as the inverse number of sequences within Hamming distance 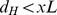, with an arbitrary but fixed 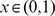:
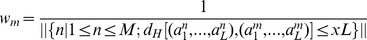
(27)for all 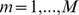. The weight equals one for isolated sequences, and becomes smaller the denser the sampling around a sequence is. Note that 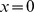 would account to removing double counts from the MSA. The total weight
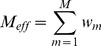
(28)can be interpreted as the effective number of independent sequences.

With these two modifications, frequency counts become
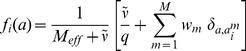
(29)

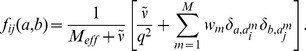
(30)Values 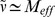 and 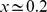 were found to work optimally across many protein families [19], we use these values. Besides these modifications, the Hopfield-Potts-model learning is performed as explained before.

### The number of independent model parameters

Amino-acid frequencies are not independent numbers. For instance, on each site 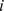, the 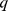 amino-acid frequencies add up to one,
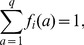
(31)and two-site distributions have single-site distributions as marginals,
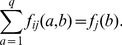
(32)As a consequence, not all of the constraints (4) and (5) are independent, and the Potts model as given in Eq. (7) has more free parameters than needed to fulfill the constraints. Families of distinct parameter values result in the same model 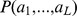 (in physics language, this corresponds to a gauge invariance: any function 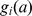 can be added to 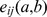 and, simultaneously, be subtracted from 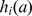, without changing the values of 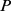). As in [19], we remove this freedom by setting
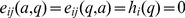
(33)for all positions 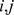 and all amino acids 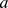. Within this setting, each choice for the parameter values corresponds to a different outcome for 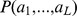. The parameters to be computed are therefore the couplings 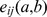 and the fields 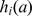 with 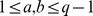 only.

An different choice for the gauge is proposed in Text S1, Section 3, and leads to quantitatively equivalent predictions for the pattern structures and the contact map.

### Mean-field theory for determining the Hopfield-Potts patterns

The MaxEnt approach underlying DCA can be rephrased in a Bayesian framework. Assume the model to be given by Eq. (7), and assume the sequences in the MSA to be independently and identically sampled from 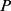. The probability of the alignment for given model parameters (couplings and fields) is then given by
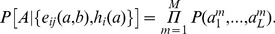
(34)Plugging in Eq. (7) and defining the log-likelihood of the model parameters given the MSA 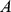, we find
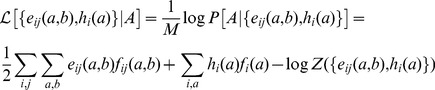
(35)One can readily see that the parameters 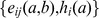 maximizing 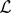 are solutions of Eqs. (4) and (5). The corresponding value for the maximum of 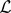 coincides with the opposite of the entropy, 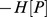, for the MaxEnt distribution given by Eq. (7).

Following the study of the Ising model case (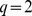) in [43], mean-field theory can be used to derive an approximate expression for the log-likelihood 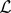 (35) when the couplings are chosen to obey Hopfield's prescription, Eq. (18). Calculations are presented in Text S1, Section 1. After optimization over the fields, we are left with the log-likelihood for the patterns only,
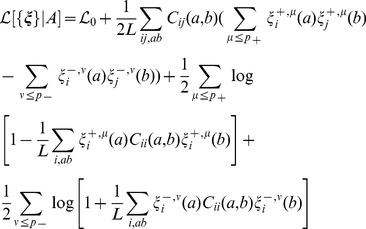
(36)


where 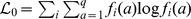. So we find the trivial result that, for 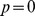 (no couplings), the log-likelihood is the negative of the sum of all single-column entropies, 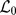. The optimal patterns, *i.e.* those optimizing the log-likelihood 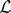 are given by Eqs. (19) and (20). The total log-likelihood corresponding to this selection reads:
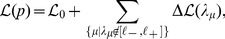
(37)where function 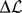 is defined in Eq. (23), and the bounds 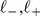 are defined in the Results Section.

The solution given in Eqs. (19) and (20) is defined up to a rotation in the pattern space, *i.e.* up to multiplication of all patterns with an indefinite orthogonal 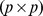
–matrix, 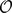, in 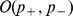. Indeed, the patterns 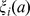 and their rotated counterparts 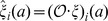 define the same set of couplings 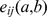 through Eq. (18). Note that this invariance is specific to the Hopfield model, and should not be mistaken for the gauge invariance of the Potts model discussed in the Results Sections. We eliminate this arbitrariness according to the following procedure, detailed in Text S1: Our selection corresponds to the case where patterns are added one after the other, starting with the best possible single pattern, followed by the second best (orthogonal to the first one when single-site correlations 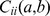 are factored out) etc.

### Contact prediction from couplings

Intuitively, residue position pairs with strong direct couplings are our best predictions for native contacts in the protein structure. To measure ‘coupling strength’, we need, however, to map the inferred 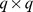 coupling matrices 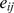 onto a scalar parameter, for each 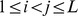. Whereas previous works on DCA have mainly used the so-called direct information [17], [19], it was recently observed that a different score actually improves the contact prediction starting from the same model parameters 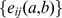 [49]. To this end, we introduce the Frobenius norm
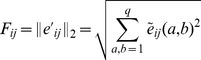
(38)of the linearly transformed coupling matrices
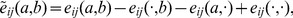
(39)where ‘⋅’ denotes average over all amino acids and the gap in the concerned position. According to the above discussion, this corresponds to another gauge of the Hopfield-Potts model, more precisely to the gauge minimizing the Frobenius norm of each coupling matrix [17]. Further more, the norm is adjusted by an *average product correction* (APC) term, introduced in [16] to suppress effects from phylogenetic bias and insufficient sampling. Incorporating also this correction, we get our final scalar score:
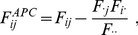
(40)where the ‘⋅’ now indicates a position average.

Sorting column pairs 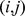 by decreasing values of 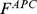 calculated using standard mean-field DCA was shown to give accurate predictions for residue contacts in various proteins, *i.e.* in the case where all possible patterns are included (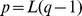) in Eq. (18). The Results Section shows how the performance in contact prediction varies when the number of patterns is 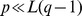.

Note that this criterion gives a coupling score to each pair of residue positions. The method itself does not provide a cutoff value for this score, below which predictions should not considered any more. Results are therefore typically provided as parametric plots depending on the number of predicted contacts as a free parameter.

## Supporting Information

Text S1 Supporting Information for From principal component to direct coupling analysis of coevolution in proteins: Low–eigenvalue modes are needed for structure prediction.
(PDF)

Code S1 Matlab code for the Hopfield-Potts inference.
(ZIP)
