# Supplementary material for: From Principal Component to Direct Coupling Analysis of Coevolution in Proteins: Low-Eigenvalue Modes are Needed for Structure Prediction
Source: PLoS Comput Biol. 2013 Aug 22;9(8):e1003176. doi: 10.1371/journal.pcbi.1003176 (PMC3749948; doi:10.1371/journal.pcbi.1003176)
Supplement: Text S1 — Supporting Information for From principal component to direct coupling analysis of coevolution in proteins: Low–eigenvalue modes are needed for structure prediction. (PDF) [file pcbi.1003176.s001.pdf]

Text S1

From principal component to direct coupling analysis of coevolution in proteins: Low–eigenvalue modes are needed for structure prediction

Supporting Information

S. Cocco, R. Monasson, M. Weigt

Equations, figures and citations included in the main article are referred to as, respectively, Eq. (X), Fig. X, and [X]. Equations, figures, and citations introduced in the Supporting Information are preceded by S, *e.g.* Eq. (S1) for the first equation below.

S1.1. MEAN-FIELD APPROXIMATION FOR THE HOPFIELD-POTTS PROBLEM

In this section, we present the details of the derivation of the log-likelihood for the Hopfield-Potts model. For a sequence $(a_1, ..., a_L)$, the Hamiltonian of the Hopfield-Potts model reads,

$$\mathcal{H} = -\frac{1}{2L}\sum_{\mu=1}^{p}\left(\sum_{i=1}^{L}\xi_i^\mu(a_i)\right)^2 - \sum_{i=1}^{N} h_i(a_i) \tag{S1}$$

cf. Eqs. (7) and (18). Note that, to lighten notations, we do not explicitly distinguish here between attractive and repulsive patterns. Attractive patterns simply correspond to real-valued patterns: $\xi_i^\mu(a) = \xi_i^{+,\mu}(a)$. Repulsive patterns have purely imaginary components in the formula above: $\xi_i^\nu(a) = \hat{\imath}\, \xi_i^{-,\nu}(a)$, with $\hat{\imath}^2 = -1$ and $\xi_i^{-,\nu}(a)$ real-valued.

Our aim is to calculate the partition function

$$\mathcal{Z} = \sum_{\{a_i|i=1,\dots,L\}} \exp(-\mathcal{H}) \ . \tag{S2}$$

The sum over the sequence can be performed once the quadratic terms in Eq. (S1) have been linearized using Hubbard-Stratonovich transformations for each μ,

$$\begin{aligned} \mathcal{Z} &= \int \prod_{\mu=1}^{p} \frac{dx^\mu}{\sqrt{2\pi/L}} \sum_{\{a_i|i=1,\dots,L\}} \exp\left\{-\frac{L}{2}\sum_{\mu=1}^{p}(x^\mu)^2 + \sum_{i=1}^{L}\left(h_i(a_i) + \sum_{\mu=1}^{p} x^\mu \xi_i^\mu(a_i)\right)\right\} \\ &= \int \prod_{\mu=1}^{p} \frac{dx^\mu}{\sqrt{2\pi/L}} \exp\left\{-\frac{L}{2}\sum_{\mu=1}^{p}(x^\mu)^2 + \sum_{i=1}^{L}\log\left(1+\sum_{a=1}^{q-1}\exp\left[h_i(a)+\sum_{\mu=1}^{p}x^\mu\xi_i^\mu(a)\right]\right)\right\} \ . \end{aligned} \tag{S3}$$

The leading contribution of the x^μ-integrations can be determined by the saddle-point approximation. The saddle points x_0^μ satisfy the equations

$$\begin{aligned} x_0^\mu &= \frac{1}{L}\sum_{ia}\xi_i^\mu(a)T_i^a \quad \text{with} \\ T_i^a &= \frac{\exp\left[h_i(a)+\sum_{\mu=1}^{p}x_0^\mu\xi_i^\mu(a)\right]}{1+\sum_{b=1}^{q-1}\exp\left[h_i(b)+\sum_{\mu=1}^{p}x_0^\mu\xi_i^\mu(b)\right]} \ . \end{aligned} \tag{S4}$$

In the above equation and in the following (unless specified explicitely) the sums over a, b run from 1 to $q-1$. The quantity T_i^a has a simple interpretation: it is equal to the marginal probability $P_i(a)$ of amino-acid a on site i. Indeed, keeping only the dominant contribution to the integral over the x^μ's in our estimate for $\mathcal{Z}$, we have

$$P_i(a) \equiv \sum_{a_k;k\neq i} P(a_1, a_2, \dots, a_L) = \frac{\partial \ln \mathcal{Z}}{\partial h_i(a)} = T_i^a \tag{S5}$$

according to definition (S4). Therefore, we have $T_i^a = f_i(a)$, the empirical frequency count computed from the sequence alignment. The fields $h_i(a)$ can therefore be computed as functions of the patterns and of the empirical frequencies through the inversion of (S4):

$$h_i(a) = \log\left(\frac{f_i(a)}{f_i(q)}\right) - \frac{1}{L}\sum_{\mu j b} \xi_i^\mu(a)\xi_j^\mu(b) f_j(b) \ . \tag{S6}$$

The saddle-point contribution to the partition function consequently reads

$$\mathcal{Z}_{SP} = \exp\left\{ -\frac{1}{2L}\sum_{\mu=1}^{p}\left[\sum_{ia}\xi_i^\mu(a) f_i(a)\right]^2 - \sum_{i=1}^{L}\log f_i(q)\right\} \ . \tag{S7}$$

As the next step, we determine the Gaussian corrections to this saddle point. To this aim we write $x^\mu = x_0^\mu + y^\mu$. Linear terms in y^μ vanish due to the saddle-point condition. To determine the quadratic terms, we need to calculate

$$\frac{\partial^2}{\partial x^\mu \partial x^\nu}\sum_i \log\left(1 + \sum_{a=1}^{q-1}\exp\left[h_i(a) + \sum_{\mu=1}^{p} x^\mu \xi_i^\mu(a)\right]\right)\Bigg|_{x_0^\mu} = \sum_i \sum_{a,b}\xi_i^\mu(a)\xi_i^\nu(b)\left[f_i(a)\delta_{a,b} - f_i(a)f_i(b)\right] \ . \tag{S8}$$

Note that in the last term, the expression $f_i(a)\delta_{a,b} - f_i(a)f_i(b)$ equals the single-site covariance matrix entry, $C_{ii}(a,b)$. Keeping only second-order terms in y^μ, we can perform the integrations and find

$$\mathcal{Z} = \frac{\mathcal{Z}_{SP}}{\sqrt{\det B}} \tag{S9}$$

with

$$B_{\mu\nu} = \delta_{\mu,\nu} - \frac{1}{L}\sum_i\sum_{a,b}\xi_i^\mu(a)\, C_{ii}(a,b)\, \xi_i^\nu(b) \ . \tag{S10}$$

Remember that Eq. (18) for the couplings is not a unique description, patterns are only determined up to an orthogonal transformation in the p-dimensional pattern space (μ space). We can therefore choose a rotation such that the matrix B becomes diagonal, i.e. such that

$$\sum_i\sum_{a,b}\xi_i^\mu(a)\, C_{ii}(a,b)\, \xi_i^\nu(b) = 0 \tag{S11}$$

for all $\mu \neq \nu$. With this choice, the determinant is readily evaluated, and we find the partition function to be

$$\log\mathcal{Z} = -\frac{1}{2L}\sum_{\mu=1}^{p}\left[\sum_{ia}\xi_i^\mu(a) f_i(a)\right]^2 - \sum_{i=1}^{L}\log f_i(q) - \frac{1}{2}\sum_{\mu=1}^{p}\log\left[1 - \frac{1}{L}\sum_{i,a,b}\xi_i^\mu(a)\, C_{ii}(a,b)\, \xi_i^\mu(b)\right] \ . \tag{S12}$$

In this expression, the partition function is given in dependence of the model parameters $h_i(a)$ and $\xi_i^\mu(a)$. To estimate these parameters from data, we have to maximize the log-likelihood given in Eq. (35). In our case it reads

$$\begin{aligned}\mathcal{L}(\{\xi_i^\mu(a), h_i(a)\}|A) \ = \ & \frac{1}{2L}\sum_{\mu,i,j,a,b}\xi_i^\mu(a)\, C_{ij}(a,b)\, \xi_j^\mu(b) + \sum_{ia} h_i(a) f_i(a) + \sum_i \log f_i(q) \\ & + \frac{1}{2}\sum_\mu \log\left[1 - \frac{1}{L}\sum_{i,a,b}\xi_i^\mu(a)\, C_{ii}(a,b)\, \xi_i^\mu(b)\right] \ . \end{aligned} \tag{S13}$$

We then eliminate the fields $h_i(a)$ from the likelihood using expression (S6), and find expression (36) for the log-likelihood $\mathcal{L}$ as a function of the patterns only.

The fact that we have chosen the specific form of Hopfield-Potts couplings instead of a general coupling matrix, does not allow us to perfectly fulfill Eq. (4) & (5), that is to perfectly fit the empirical two-site correlations by the model. Instead, the optimization has to be done with respect to the pattern entries $\xi_i^\mu(a)$. To simplify the notation, we introduce vectors

$$u_{ia}^\mu = \frac{1}{\sqrt{L}}\sum_{b=1}^{q-1} D_i(a,b)\xi_i^\mu(b) \tag{S14}$$

with D_i defined in Eq. (11) as the square root of the $(q-1)\times(q-1)$ single-site correlation matrix C_{ii}. The norm of these vectors is denoted as $U^\mu = [\sum_{ia}(u^\mu_{ia})^2]^{1/2}$. Replacing ξ by u, and using definition (10), the log-likelihood becomes

$$\mathcal{L}(\{u^\mu_{ia}\}|A) = \sum_i \sum_{a=1}^{q} f_i(a)\log f_i(a) + \frac{1}{2}\sum_{\mu,i,j,a,b} u^\mu_{ia}\,\Gamma_{ij}(a,b)\,u^\mu_{jb} + \frac{1}{2}\sum_\mu \log\left[1-(U^\mu)^2\right] \ . \tag{S15}$$

Maximization with respect to u^μ_{ia} yields the equation

$$\sum_{jb}\Gamma_{ij}(a,b)u^\mu_{jb} = \frac{u^\mu_{ia}}{1-(U^\mu)^2} \ , \tag{S16}$$

i.e. $(u^\mu_{ia})^{a=1\dots q-1}_{i=1\dots L}$ is an eigenvector of Γ with eigenvalue $\lambda_\mu = 1/[1-(U^\mu)^2]$. Introducing the normalized vectors

$$v^\mu_{ia} = \frac{\sqrt{L}}{U^\mu}u^\mu_{ia} \ , \tag{S17}$$

we finally find Eqs. (19) and (20) for the maximum-likelihood pattern. We correctly find back that attractive patterns correspond to eigenvalues λ larger than unity, while patterns attached to eigenvalues smaller than one are purely imaginary, and thus correspond to repulsive patterns.

The final expression for the likelihood reads therefore

$$\mathcal{L}(A) = \sum_i \sum_{a=1}^{q} f_i(a)\log f_i(a) + \frac{1}{2}\sum_\mu \left[\lambda_\mu - 1 - \log\lambda_\mu\right] \ , \tag{S18}$$

and we have to select the p eigenvalues λ_μ maximizing this expression.

From a statistical physics perspective, it is important to emphasize that the relationship between the maximum entropy principle and principal component analysis holds within the mean-field approximation only. The solution of the inverse Hopfield-Potts model presented in this paper neglects corrections to the mean-field approximation, which would appear as finite-L corrections to the expressions of the patterns. Those corrections were computed in [39] for the Ising case ($q=2$), and we expect that the change in the patterns are non-linear functions of the eigenmodes components, including also contributions from the eigenmodes in the bulk of the Marcenko-Pastur spectrum (with eigenvalues close to unity). Furthermore it would be interesting to see how computational techniques developed to infer the coupling matrix e going beyond mean-field could be extended to implement the low-rank constraint intrinsic to the Hopfield model.

S1.2. SPECTRUM OF Γ FOR CONSERVED SEQUENCES

In this appendix, we derive the spectrum of the correlation matrix Γ, cf. Eq. (10), for the case of L conserved sites, i.e. for the case of M exactly repeated sequences. Without loss of generality, we assume the conserved value to correspond to the first residue, i.e. $a^m_i \equiv 1$ for all $i=1,...,L$ and all $m=1,...,M$. Note that in this case the final result does not depend on M, since all weights in Eq. (27) become equal to $1/M$, and the effective number of sequences is $M_{eff}=1$, cf. Eq. (28).

Introducing a pseudocount $\nu = \tilde{\nu}/(M_{eff}+\tilde{\nu})$, the frequency counts become $(i\neq j)$

$$\begin{aligned} f_i(a) &= (1-\nu)\delta_{a,1} + \frac{\nu}{q} \\ f_{ii}(a,b) &= \left[(1-\nu)\delta_{a,1} + \frac{\nu}{q}\right]\delta_{a,b} \\ f_{ij}(a,b) &= (1-\nu)\delta_{a,1}\delta_{b,1} + \frac{\nu}{q^2} \ , \end{aligned} \tag{S19}$$

and the connected correlations read

$$\begin{aligned} C_{ii}(a,b) &= \frac{\nu(1-\nu)}{q^2}(1-q\delta_{a,1})(1-q\delta_{b,1}) - \frac{\nu}{q^2}(1-\delta_{a,b}) \\ C_{ij}(a,b) &= \frac{\nu(1-\nu)}{q^2}(1-q\delta_{a,1})(1-q\delta_{b,1}) \end{aligned} \tag{S20}$$

Due to the rescaling in Γ, constant prefactors are not important, and for simplicity of notation we can equivalently work with the matrix

$$\tilde{C} = \frac{q^2}{\nu(1-\nu)} C \tag{S21}$$

instead of the true covariances. Entries read

$$\begin{aligned} \tilde{C}_{ii}(a,b) &= (1-q\delta_{a,1})(1-q\delta_{b,1}) - \frac{1}{1-\nu}(1-\delta_{a,b}) \\ \tilde{C}_{ij}(a,b) &= (1-q\delta_{a,1})(1-q\delta_{b,1}) \ . \end{aligned} \tag{S22}$$

If we have a close look to the off-diagonal $i \neq j$–blocks of size $(q-1)\times(q-1)$, we always find the same matrix,

$$\tilde{C}_{ij} = \begin{pmatrix} (1-q)^2 & (1-q) & \cdots & (1-q) \\ (1-q) & 1 & \cdots & 1 \\ \vdots & \vdots & & \vdots \\ (1-q) & 1 & \cdots & 1 \end{pmatrix} . \tag{S23}$$

Note that this matrix is of rank one, and can be written as

$$\tilde{C}_{ij} = |\tilde{x}\rangle\langle\tilde{x}| \tag{S24}$$

with

$$\langle\tilde{x}| = (1-q, 1, \cdots, 1) \ . \tag{S25}$$

For the case of fully conserved sequences, the rescaled covariance matrix assumes a characteristic block-diagonal form

$$\Gamma = \begin{pmatrix} \mathbb{I} & \Gamma_{12} & \Gamma_{12} & \cdots \\ \Gamma_{12} & \mathbb{I} & \Gamma_{12} & \cdots \\ \Gamma_{12} & \Gamma_{12} & \mathbb{I} & \cdots \\ \vdots & \vdots & \vdots & \end{pmatrix} \tag{S26}$$

with a total of $L \times L$ blocks of size $(q-1)\times(q-1)$, and with $\mathbb{I}$ being the unit matrix. All off-diagonal blocks are identical and given by

$$\Gamma_{12} = \left(\tilde{C}_{11}\right)^{-\frac{1}{2}} \tilde{C}_{12} \left(\tilde{C}_{22}\right)^{-\frac{1}{2}} \ . \tag{S27}$$

If we further use $\tilde{C}_{22} = \tilde{C}_{11} = \tilde{C}_{11}^t$ and Eq. (24), we find that

$$\Gamma_{12} = \left[\left(\tilde{C}_{11}\right)^{-\frac{1}{2}} |\tilde{x}\rangle\right] \left[\left(\tilde{C}_{11}\right)^{-\frac{1}{2}} |\tilde{x}\rangle\right]^t \tag{S28}$$

has also rank one.

If we would know the non-trivial eigenvector $|y\rangle$ of Γ_{12} with eigenvalue λ, we could immediately read of the full spectrum of Γ. It is easy to see, that the L-fold concatenation of $|y\rangle$ with itself is an eigenvector of Γ,

$$\Gamma \begin{pmatrix} |y\rangle \\ |y\rangle \\ \vdots \\ |y\rangle \end{pmatrix} = [1+(L-1)\lambda] \begin{pmatrix} |y\rangle \\ |y\rangle \\ \vdots \\ |y\rangle \end{pmatrix} . \tag{S29}$$

Further more, the $L-1$ vectors (with 0_{q-1} being a vector of $q-1$ zero-elements)

$$\begin{pmatrix} |y\rangle \\ -|y\rangle \\ 0_{q-1} \\ \vdots \\ 0_{q-1} \end{pmatrix} , \begin{pmatrix} 0_{q-1} \\ |y\rangle \\ -|y\rangle \\ \vdots \\ 0_{q-1} \end{pmatrix} , \cdot \begin{pmatrix} 0_{q-1} \\ \vdots \\ 0_{q-1} \\ |y\rangle \\ -|y\rangle \end{pmatrix} , \tag{S30}$$

span an eigenspace for eigenvalue $1-\lambda$. For any vectors $|z_1\rangle, ..., |z_L\rangle$ with $\Gamma|z_i\rangle = 0_{q-1}$ for all $i = 1, ..., L$, we find

$$\Gamma \begin{pmatrix} |z_1\rangle \\ |z_2\rangle \\ \vdots \\ |z_L\rangle \end{pmatrix} = \begin{pmatrix} |z_1\rangle \\ |z_2\rangle \\ \vdots \\ |z_L\rangle \end{pmatrix} . \tag{S31}$$

Doing so, we find that Γ has one non-degenerate eigenvalue $1+(L-1)\lambda$, one $(L-1)$-fold degenerate eigenvalue $1-\lambda$, and one $L(q-2)$-fold degenerate eigenvalue equal to one. This observation (together with the fact that real alignments have many strongly, but not perfectly conserved sites) explains already the existence of a peak in the spectrum of Γ, which is centred around one. In addition, the inverse participation ratio (IPR) of the eigenvectors (S30) is half the IPR of $|y\rangle$, whereas the IPR of eigenvector (S29) is only $1/L$-times the IPR of $|y\rangle$. This explains the stronger localization of eigenvectors in the small tail of the spectrum, as compared to the eigenvectors of large eigenvalues. Since the transformation Eqs. (19,20,21) are local in the sites $i = 1, .., L$, these localization properties are automatically transfered to the final Hopfield patterns. Note also that the structure of the vectors (and hence the Hopfield patterns) is coherent with their attractive/repulsive nature: The simple repeat vector corresponds to an eigenvalue larger than one, i.e. to an attractive pattern. The repeat structure implies that residue values tend to behave equally, as the conserved value ($a = 1$ in our calculation). The basis eigenvectors for eigenvalues smaller than one, and thus also the repulsive patterns, have entries of opposite sign in the two sites with non-zero entries. In consequence, the two sites tend to avoid directions where they behave in an opposite way, in particular where one variable takes the conserved value, and the other any other value.

The task of determining the $L(q-1)$ eigenvalues of Γ is thus broken down to the task of determining the only non-trivial eigenvalue λ of the $(q-1)\times(q-1)$-matrix Γ_{12}. And since there is only one non-zero eigenvalue, it equals the trace of Γ_{12}. Exploiting the cyclicity of the trace of matrix products, we find

$$\lambda = \mathrm{tr}\left(\tilde{C}_{11}^{-\frac{1}{2}}|\tilde{x}\rangle\langle\tilde{x}|\tilde{C}_{11}^{-\frac{1}{2}}\right) = \langle\tilde{x}|C_{11}^{-1}|\tilde{x}\rangle . \tag{S32}$$

To invert C_{11}, we calculate its eigenvalue decomposition. It has $q-3$ linearly independent eigenvectors of the form $(0,1,-1,0,...,0)^t, (0,0,1,-1,0,...,0)^t, ..., (0,...,0,1,-1)^t$ with the $(q-3)$-fold degenerate eigenvalue $q/(1-\nu)$. These vectors are orthogonal to $|\tilde{x}\rangle$ and therefore do not contribute to Eq. (32). The other two eigenvectors are orthogonal to these $q-3$ vectors, i.e. they are of the form $(a,1,...,1)$. The eigenvalue equations read

$$\begin{aligned} a\left[(1-q)^2 + \frac{q-1}{1-\nu}\right] + (q-2)\frac{\nu q - q - \nu}{1-\nu} &= \Lambda a \\ a\frac{\nu q - q - \nu}{1-\nu} + \frac{q-\nu}{1-\nu} - (q-3)\frac{\nu}{1-\nu} &= \Lambda . \end{aligned} \tag{S33}$$

Elimination of a leads to a quadratic equation in Λ, with solutions

$$\Lambda_{1,2} = \frac{q^2(1-\nu)+q\nu+\nu}{2(1-\nu)} \pm \frac{1}{1-\nu}\sqrt{\frac{[q^2(1-\nu)+q\nu+\nu]^2}{4} - q^2(1-\nu) - q\nu} , \tag{S34}$$

and, using the second of Eqs. (33), we also find

$$a_{1,2} = \frac{\Lambda_{1,2}(1-\nu)+q\nu-q-2\nu}{q\nu-q-\nu} . \tag{S35}$$

This allows us to finally go back to Eq. (32),

$$\lambda = \langle\tilde{x}|C_{11}^{-1}|\tilde{x}\rangle = \sum_{s=0,1}\frac{1}{\Lambda_s}\frac{\langle\tilde{x}|a_s,1,...,1\rangle^2}{||(a_s,1,...,1)||^2} = \sum_{s=0,1}\frac{1}{\Lambda_s}\frac{[(1-q)a_s+q-2]^2}{a_s^2+q-2} . \tag{S36}$$

Note that, even if the covariances vanish for $\nu\to 0$, these eigenvalues have a well-defined limit. For $\nu = 0$ we find

$$\begin{aligned} \Lambda_{1,2} &= \frac{q^2}{2} \pm \sqrt{\frac{q^4-4q^2}{4}} \\ a_{1,2} &= 1 - \frac{q}{2} \pm \sqrt{\frac{q^2-4}{4}} . \end{aligned} \tag{S37}$$

For the protein case $q = 21$ we find thus the following numerical values

$$\begin{aligned} \lambda(\nu = 0) &= 0.9524 \\ \lambda(\nu = \frac{1}{2}) &= 0.9091 \\ \lambda(\nu = 1) &= 0\ . \end{aligned} \qquad (S38)$$

Note that the value does not change much between $\nu = 0$ (no pseudocount) and $\nu = 1/2$, i.e. $\tilde{\nu} = M_{eff}$. Note also that the numerical values for the support of the spectrum, $[1-\lambda, 1+(L-1)\lambda] \simeq [0.09, 1+0.91(L-1)]$, are consistent with the empirically found spectra.

S1.3. ALTERNATIVE GAUGE FOR THE HOPFIELD-POTTS MODEL

In the Potts model defined in Eq. (7), changes of the couplings $e_{ij}(a,b) \to e_{ij}(a,b) + g_i(a)$ can be compensated by corresponding changes of the field $h_i(a) \to h_i(a) - g_i(a)$, see Methods. In the main paper we have removed this gauge invariance by specializing to couplings matrices $e_{ij}(a,b)$ where the qth row $(a = q)$ and column $(b = q)$ are equal to zero for every pair of sites $i < j$. In this appendix we consider another, natural choice of the gauge and show that this choice gives essentially the same results for the localization properties of the patterns and for the contact map predictions.

We define from the multiple sequence alignment the full $(L \times q)$-dimensional covariance matrix,

$$C^*_{ij}(a,b) = f_{ij}(a,b) - f_i(a) f_j(b)\ , \qquad (S39)$$

and its associated Pearson $(L \times q)$–dimensional correlation matrix,

$$\Gamma^*_{ij}(a,b) = \frac{f_{ij}(a,b) - f_i(a) f_j(b)}{\sqrt{f_i(a)\, f_j(b)}} \qquad (S40)$$

These matrix has L zero eigenvalues, because on each site the probabilities over all possible $q = 21$ amino-acid or gap symbols sums up to 1:

$$\sum_{b=1}^{q} f_j(b) = 1 \quad \Rightarrow \quad \sum_{b=1}^{q} \Gamma^*_{ij}(a,b)\, \sqrt{f_j(b)} = 0\ , \qquad \forall\ i, a. \qquad (S41)$$

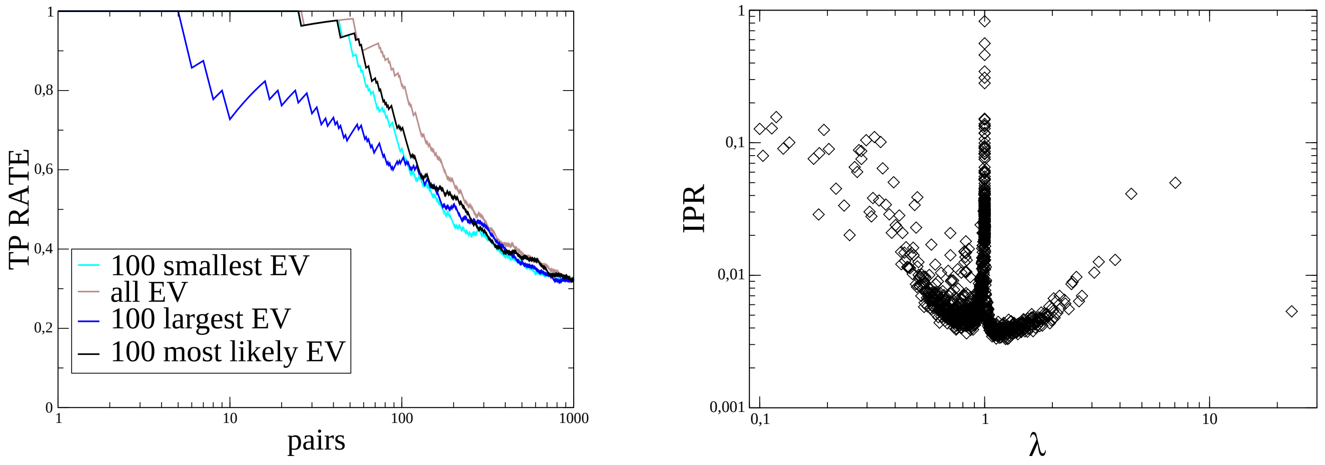

Figure S1: **Results for PF00014 in the gauge orthogonal to the vectors** $f_i(a)$**:** *(left)* TP rate for the contact prediction using the 100 most likely Hopfield patterns (black) and the patterns corresponding to the 100 smallest, respectively largest eigenvalues (cyan, resp. blue). *(right)* The inverse participation ratio of the Hopfield patterns as a function of the corresponding eigenvalue λ.

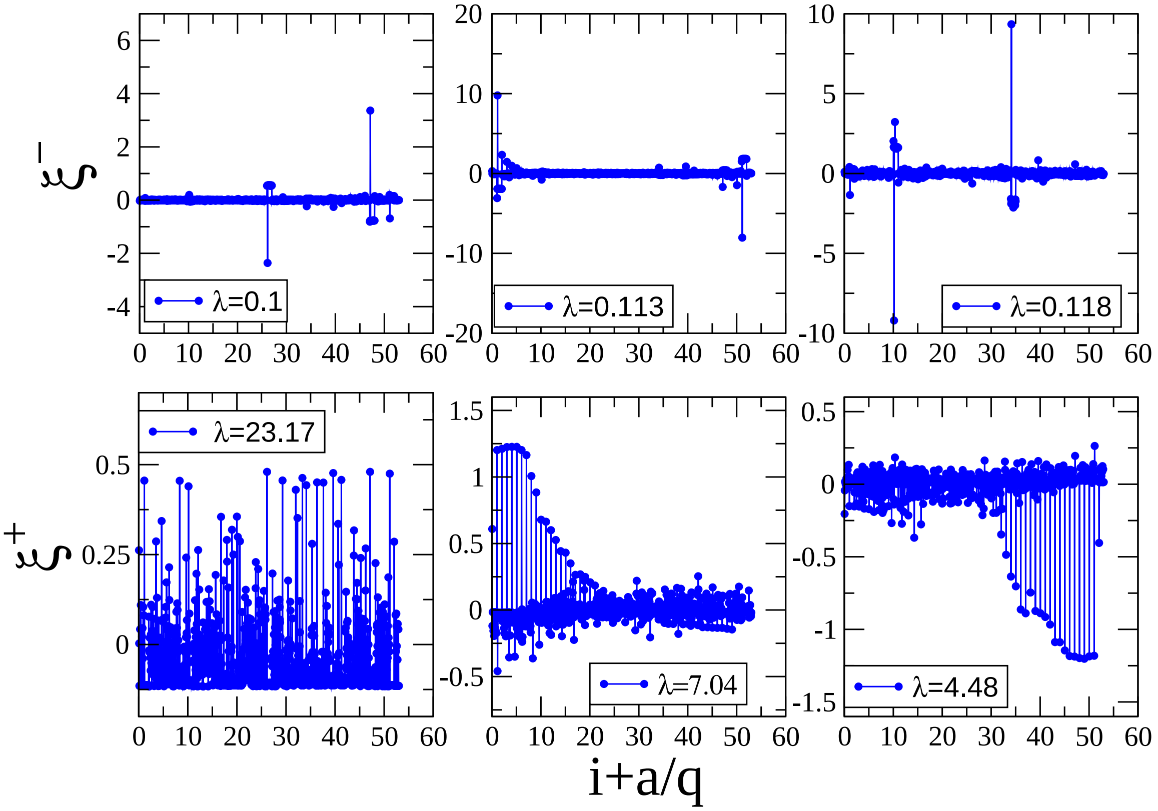

Figure S2: **Results for PF00014 in the gauge orthogonal to the vectors $f_i(a)$.** *(top):* first, third and fourth lowest-eigenvalue repulsive patterns, with inverse participation ratios $0.13, 0.13, 0.15$ respectively (the second lowest-eigenvalue pattern has IPR equal to 0.08). *(bottom):* top three attractive patterns corresponding to the largest three eigenvalues, with inverse participation ratios equal to $0.005, 0.05, 0.04$ respectively.

To remove those L zero eigenmodes we project Γ^* onto the $(L\times(q-1))$–dimensional space orthogonal to the vectors of components $\sqrt{f_i(a)}$. The projection matrix, which we call $\mathbf{t}^*$, can be built as follows: For each site i, we apply the Gram-Schmidt orthogonalization procedure, and find a $q\times(q-1)$ matrix t_i^*, whose transpose $(t_i^*)^\dagger$ projects onto the subspace orthogonal to the q-dimensional vector $f_i(a), a=1,\dots,q$. Gathering the L block t_i^* we obtain the block-diagonal matrix $\mathbf{t}^*$. We then compute the $(L\times(q-1))$–dimensional projected Pearson correlation matrix:

$$\Gamma_P = (\mathbf{t}^*)^\dagger\ \Gamma^*\ \mathbf{t}^* \tag{S42}$$

The matrix Γ_P is then diagonalized, with eigenvectors $(v_P)^\mu_{i,a}$ (with squared norms equal to $L(q-1)$) and eigenvalues $(\lambda_P)^\mu$. It is easy to check that

$$\sum_i \sum_{a=1}^{q-1} (\Gamma_P)_{ii}(a,a) = \sum_i \sum_{a=1}^{q} \Gamma^*_{ii}(a,a) = \sum_i \sum_{a=1}^{q} (1-f_i(a)) = L(q-1)\ . \tag{S43}$$

Therefore, though the eigenvalues λ_P^μ are different from the eigenvalues λ^μ obtained with the gauge chosen in the main paper, their average value is still equal to unity.

Repeating the mean-field calculation of Supporting Information Section S1.1, we compute the patterns within the MaxEnt principle, with the results

$$\xi_i^\mu(a) = \sqrt{1-\frac{1}{\lambda_P^\mu}}\,\frac{v^\mu_{i,a}}{\sqrt{f_i(a)}} \tag{S44}$$

where

$$\mathbf{v}^\mu = \mathbf{t}^*\ (\mathbf{v}_P)^\mu\ , \tag{S45}$$

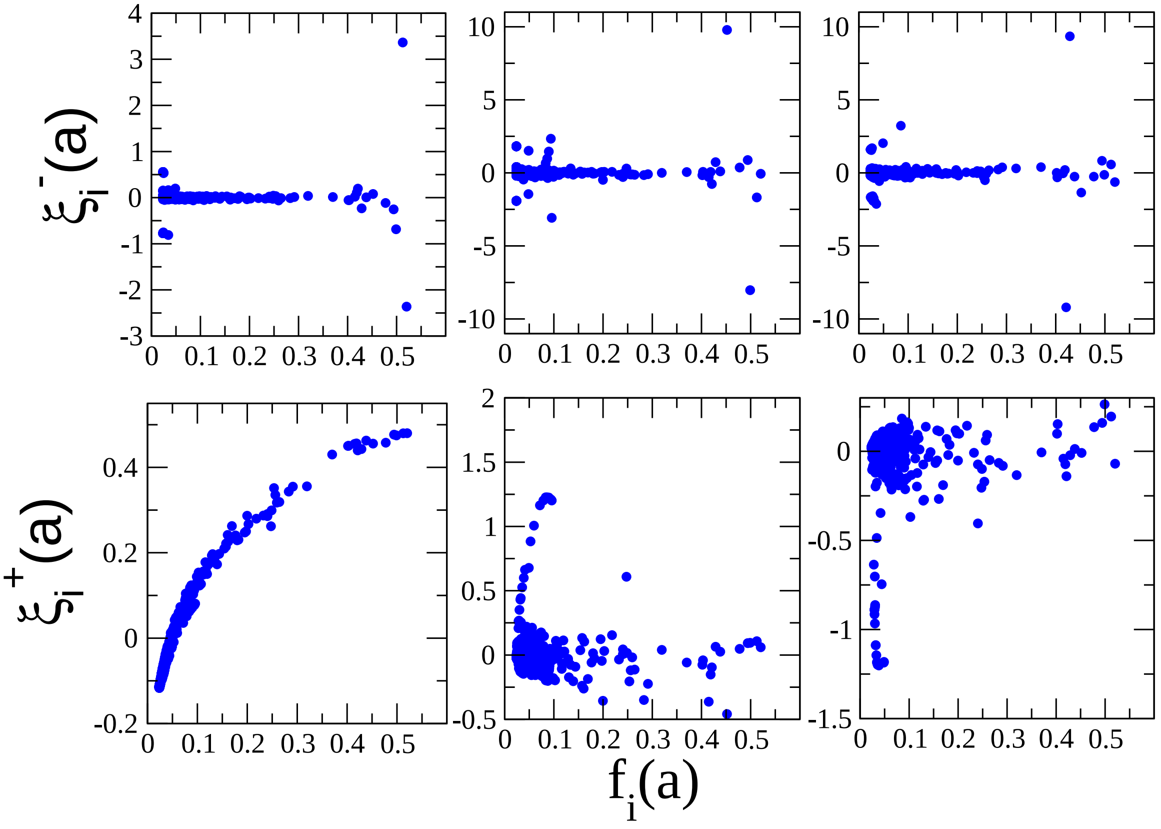

Figure S3: **Results for PF00014 in the gauge orthogonal to the vectors $f_i(a)$.** Components of the most repulsive (*upper panels)* and most attractive (*lower panels*) patterns, $\xi_i^\mu(a)$, vs. amino-acid frequencies, $f_i(a)$. Note that due to the pseudocount, a completely conserved aminoacid has frequency $f_i(a) = (M_{eff} + \tilde{\nu}/q)/(M_{eff} + \tilde{\nu}) \simeq 0.52$ (for our pseudocount $\tilde{\nu} = M_{eff}$).

is the expression of the μth eigenvector of Γ_P projected back in the original space. This in turn defines the $(L \times q)$–dimensional coupling matrix e^* through Eq. (18). Note that in the new gauge we have

$$\sum_{i,a} \xi_i^\mu(a)\ f_i(a) = 0\ , \tag{S46}$$

according to Eqs. (41) and (44). Hence, the patterns are orthogonal to the vector expressing local residue conservations.

Results for the inverse participation ratio (IPR) of the patterns and the contact map predictions are shown in Fig. S1. Even if the eigenvalues are not exactly the same, the behavior of the IPR as a function of the eigenvalue and the quality of the contact map prediction is very similar to what we obtained in the main paper (See Fig. 2 and Fig. 6). Furthermore, we show in Fig. S2, the three patterns attached to the three lowest eigenvalues. We observe that those patterns are highly localized, on the same sites as the ones found with the previous gauge, compare to Fig. 3.

As a conclusion our main results on the localization of the patterns and on their relevance to contact map prediction are independent of the choice of the Potts model gauge. The reason is that for both gauges the Pearson correlation matrix can be written as $M^\dagger\ C^*\ M$ where M is a block-diagonal matrix. Hence, while the non-zero components of the localized eigenvectors are affected by the choice of M, their supports are not.

We compare in Fig. S3 the pattern components $\xi_i^\mu(a)$ to the amino-acid (and gap) frequencies, $f_i(a)$ (with the pseudo-count). We observe that for the most attractive pattern (largest value of λ), components are monotonous functions of the residue conservation. The next two attractive patterns, which are due to the presence of repeated gaps at the extremities of the sequences, are not correlated with conservation. As for repulsive patterns, we find that the few large components correspond to strongly conserved residues. However, the correlation between conservation

and component amplitude is not monotonous as in the case of the most attractive patterns: most conserved residues may be attached to very weak patterns components.

S1.4. LIST OF PROTEIN FAMILIES

We hereafter give the list of the 15 families used to validate our approach in Fig. 6 of the main paper, see [30]:

Protein (PDB ID)	Fold	Domain Length	Name	Pfam Domain	Organism
3nnr	α	53	TetR-family transcriptional regulator	TerR_N	*Marinobacter aquaeolei*
1or7	α	70	RseA	Sigma70 region 2	*Escherichia coli*
3df8	α	91	Possible HxlR family transcriptional factor	HxlR	*Thermoplasma volcanium*
1oap	α/β	98	Peptidoglycan associated lipoprotein PAL (Periplasmic domain)	OmpA	*Escherichia coli*
3d7i	α	98	Oxygen detoxification CMD protein	CMD	*Methanococcus jannaschii*
2gj3	α/β	118	Transcriptional regulation sensor protein NifL	PAS	*Azotobacter vinelandii*
3ddv	β	139	Transcriptional regulator GntR family	UTRA	*Enterococcus faecalis*
3nkh	α	187	Integrase MRSA strain	Phage integrase	*Staphylococcus aureus*
1jft	α	54	Purine repressor PurR (N-terminal)	LacI	*Escherichia coli*
3f52	α	57	Gene regulator ClgR	HTH_3	*Corynebacterium glutamicum*
1kgs	α/β	112	Transcription factor DrrD	Receiver domain (Response regulator)	*Thermotoga maritima*
3nyy	β	112	Putative glycyl-glycine endopeptidase lytM	Peptidase_M23	*Ruminococcus gnavus*
3fwz	α/β	116	Inner membrane protein ybaL	TrkA_N	*Escherichia coli*
3fms	α	120	GntR transcriptional regulator	GntR	*Thermotoga maritima*
3bvp	α/β	133	N-terminal Catalytic Domain of TP901-1 Integrase	Resolvase	*Lactococcus phage TP901-1*

S1.5. SUPPLEMENTARY RESULTS FOR THE PROTEIN FAMILIES

S1.5.1. Family PF00014

Hereafter we report further results on protein family PF00014, not shown in the main paper. In Fig. S4 we plot the pattern components $\xi_i(a)$ shown in Fig. 3 vs. $|f_i(a) - f_i(q)|$, that is the amino-acid frequencies (with reweighting and pseudo-count), from which we have subtracted the frequency of the qth amino-acid discarded by the gauge. The

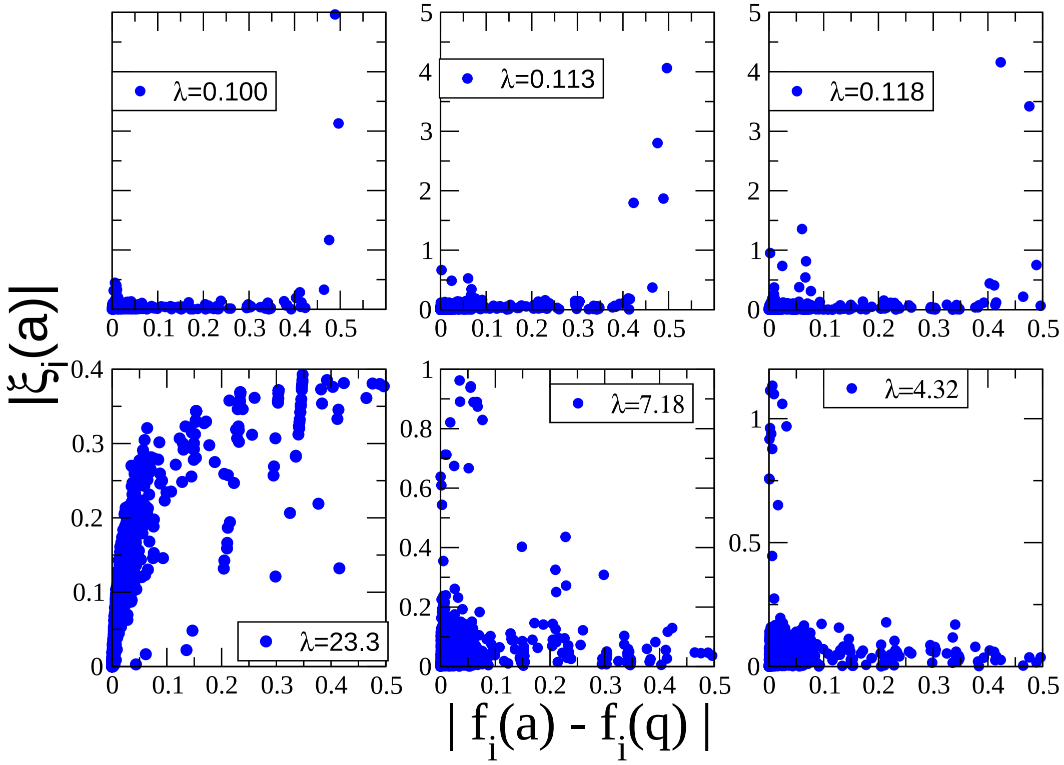

Figure S4: **Pattern components vs. residues frequencies in PF00014** for the top three repulsive (*upper panels*) and attractive (*lower panels*) patterns.

correlation between $\xi_i(a)$ and $f_i(a)$ for the top attractive pattern shows that this pattern essentially identifies the most conserved sites. On the contrary the attractive patterns related to repeated gaps at the beginning and at the end of the sequence are not conserved. We also find that the sites and amino acids on which the repulsive patterns are localized are among the most conserved on the sequence alignments. These results are equivalent to the ones found with the gauge orthogonal to $f_i(a)$, see Section S1.3.

Figure 4 in the main text suggests that some sites attached to the largest couplings of the Hopfield-Potts model, from which contacts are predicted, are conserved, while others are not. To further investigate the existence or the absence of relationship between conservation and contacts we have used the Consurf server, designed to identify functional regions in proteins (http://consurf.tau.ac.il/; Ashkenazy H, Erez E, Martz E, Pupko T, Ben-Tai N, 2010). Figure SI-5 compare site conservation, defined as the frequency $f_i(a^*) = \max_a f_i(a)$ of the most frequent amino acid a^* in position i, with the score computed with ConSurf for the PF00014 family (both conservation measures determined for the same domain-family MSA). We observe that the agreement is very good: highly conserved sites with our measure corresponds to low ConSurf scores as expected. In addition we color in red all the sites i attached to at least one of the 30 top predicted residue-residue pairs (with residue separation $|i-j| \geq 5$ along the primary sequence as in the main text), i.e. to the 30 strongest couplings of the Hopfield-Potts model with $|i-j| \geq 5$. These red points are scattered all over the plot, signaling that both more conserved and very variable sites are equally detected as strongly coupled in co-evolution. There exists no obvious correlation between predicted contacts and site conservations.

To study the amplitude of couplings e and how it depends on the number of selected patterns, p, we calculate the fraction of couplings larger than X in absolute value:

$$\phi(X) = \frac{2}{L(L-1)(q-1)^2} \sum_{i<j,a,b} \mathbf{1}_{|e_{ij}(a,b)|>X} \ . \tag{S47}$$

Note that diagonal couplings ($e_{ii}(a,b)$) are omitted in the calculation of ϕ. Results are shown in SI-Fig.6. We observe that the coupling matrix corresponding to DCA (all patterns are selected) is less 'sparse' than the coupling matrix defined by the Hopfield-Potts model. More precisely the Hopfield-Potts matrix include many small couplings,

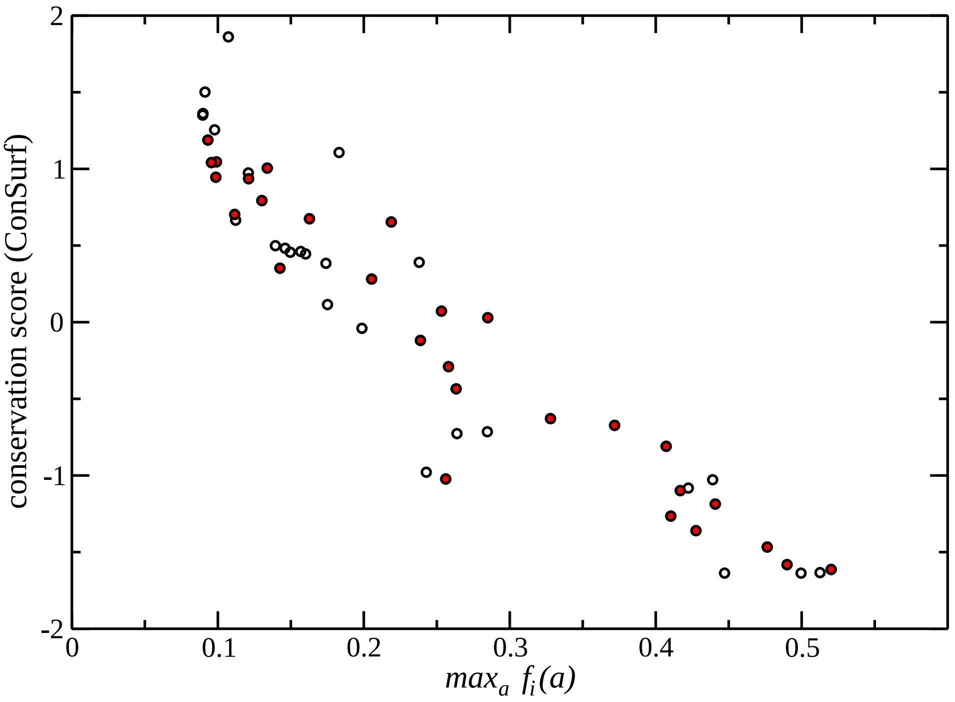

Figure S5: **Comparison of conservation scores for the PF00014 family.** Shown is a scatter plot of conservation as measured naturally in our framework by the frequency of occurrence of the most abundant amino acid of each position vs. the standard ConSurf score (which takes negative values for conserved residues), both measures are very coherent with each other. Further more, red dots correspond to positions contained in at least one out of the 30 strongest coupled residue pairs in our model (with sequence separation of at least five positions), showing that strong co-evolution is detectable for conserved and variable residues.

compared to the DCA matrix. For instance, about 1% of the Hopfield-Potts couplings for $p = 100$ patterns are larger than 0.8, while about 10% of the DCA couplings are larger than this threshold.

S1.5.2. Families PF00072 & PF00071

For each one of the two families PF00072 and PF00071, we show the localization and spectral properties of the eigenmodes of the correlation matrices, our contact predictions, the structure of the most attractive and repulsive patterns, the comparison with the residue conservation, the most conserved sites and the pairs of interacting sites on the 3D folds.

The results of the analysis of PF00072 and PF00071 are analogous to the ones found for PF00014. In particular,

- Most repulsive patterns are strongly localized (see SI-Fig. 7, SI-Fig.11 and SI-Fig.8, SI-Fig.12;
- Repulsive patterns alone give very good contact predictions (see Fig. S7, Fig. S11);
- The top attractive pattern is extended over the whole sequence, and the second and third attractive patterns correspond to repeated gaps on the edges of the sequence. Moreover, in Fig. S9 and Fig. S13, we show that the most attractive pattern components are strongly correlated with the residues frequencies, and that the most repulsive pattern have few, large components on conserved residues.
- The top attractive pattern define connected regions of conserved residues on the 3D fold, see left panels in Fig. S10 and Fig. S14. Many of the contacts predicted by our approach (red links in the right panels) are between not-conserved sites.

S1.5.3. Performance of the Hopfield-Potts model for small MSA accross 15 families

We have shown in Fig. 8 that the Hopfield-Potts model is able to predict contacts for the PF00014 domain even with very short MSA, contrary to the usual DCA approach. To check the generality of this statement we show in Fig. S15 the TP rates, averaged over the 15 protein families in the Table of Section S1.4, for reduced MSA sizes of $M = 1000$ (black lines) and $M = 50$ (red lines) sequences. For each value of M, of p, and for each family 100 random sub-MSAs were generated by picking up uniformly at random a subset of M sequences in the available MSA for the family. Whereas for large M it is optimal to use full mean-field DCA (i.e. $p = L(q-1)$), a clear optimum at an intermediate value of p, here $p = 32$, is observed for small M. Larger MSA lead to a larger optimal value for p, smaller MSA to a smaller optimal value. The actual value depends also on the family and the (random) realization of the sub-MSA.

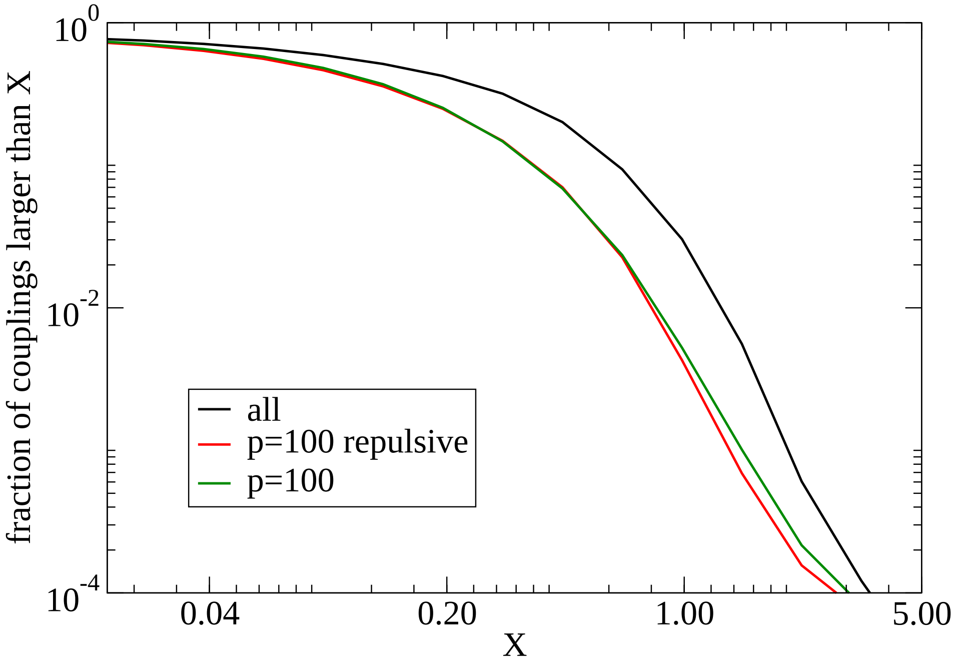

Figure S6: **Sparsity of couplings for the PF00014 family.** The curve shows the fraction of couplings $e_{ij}(a,b)$ larger (in absolute value) than X as a function of X. Results are shown for the top $p = 100$ repulsive patterns (red), for the $p = 100$ patterns contributing most to the log-likelihood ($p_- = 58, p_+ = 42$), and when all patterns are included (DCA, black curve).

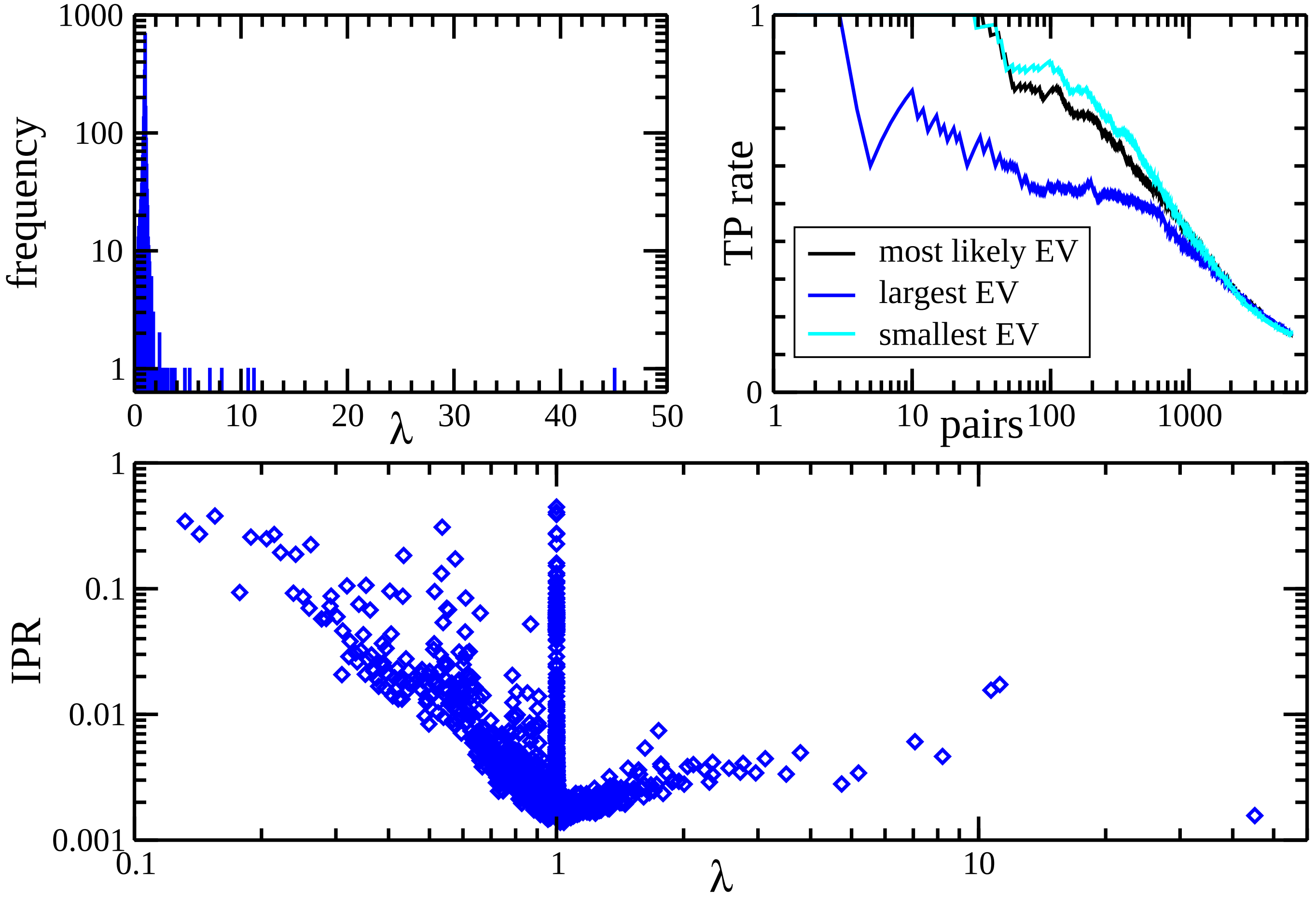

Figure S7: **Localization and contact prediction for PF00072.** *(Upper left)* The spectral density as a function of the eigenvalues λ, note the existence of few very large eigenvalues, and a pronounced peak in $\lambda = 1$. *(upper right)* TP rate for the contact prediction using the 100 most likely Hopfield patterns (black), and the patterns corresponding to the 100 smallest resp. largest eigenvalues (red resp. blue). *(lower panel)* The inverse participation ratio of the Hopfield patterns as a function of the corresponding eigenvalue λ.

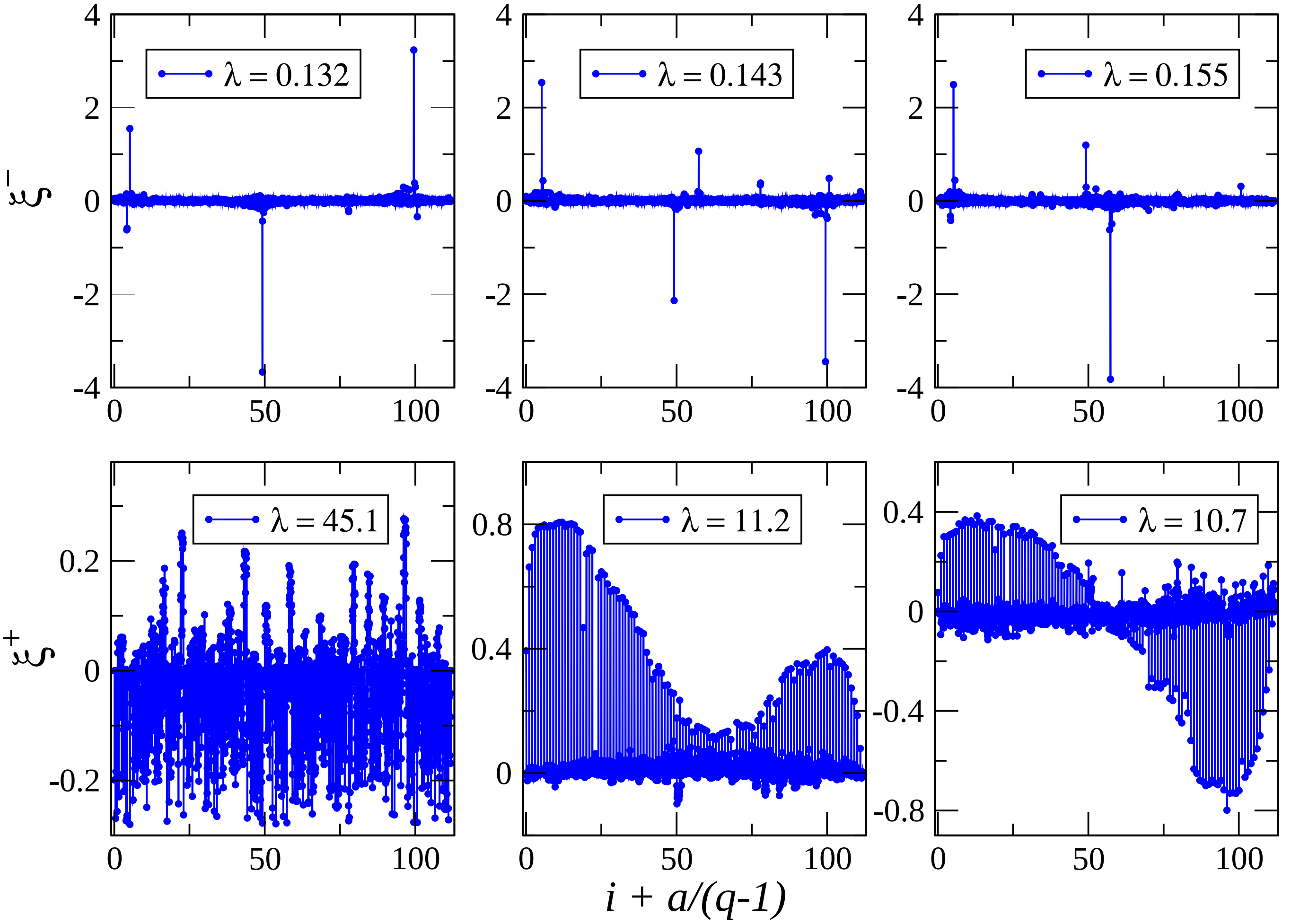

Figure S8: **Attractive and repulsive patterns for PF00072.** *(Upper panels)* The most repulsive patterns (corresponding to the first, second and third smallest eigenvalues) are strongly localized over pairs of sites with inverse participation ratios $0.34, 0.27, 0.38$ respectively. *(lower panels)* Shown are the most attractive patterns (corresponding to the three largest eigenvalues); the top pattern is extended with inverse participation ratio 0.001, while the second and third patterns have essentially non-zero components over the gap symbols only concentrated on the edges of the sequences (inverse participation ratio 0.017,0.015 respectively). Note the x-coordinates $i + a/(q-1)$; its integer part is the site index, i, and the fractional part multiplied by $q-1$ is the residue value, a.

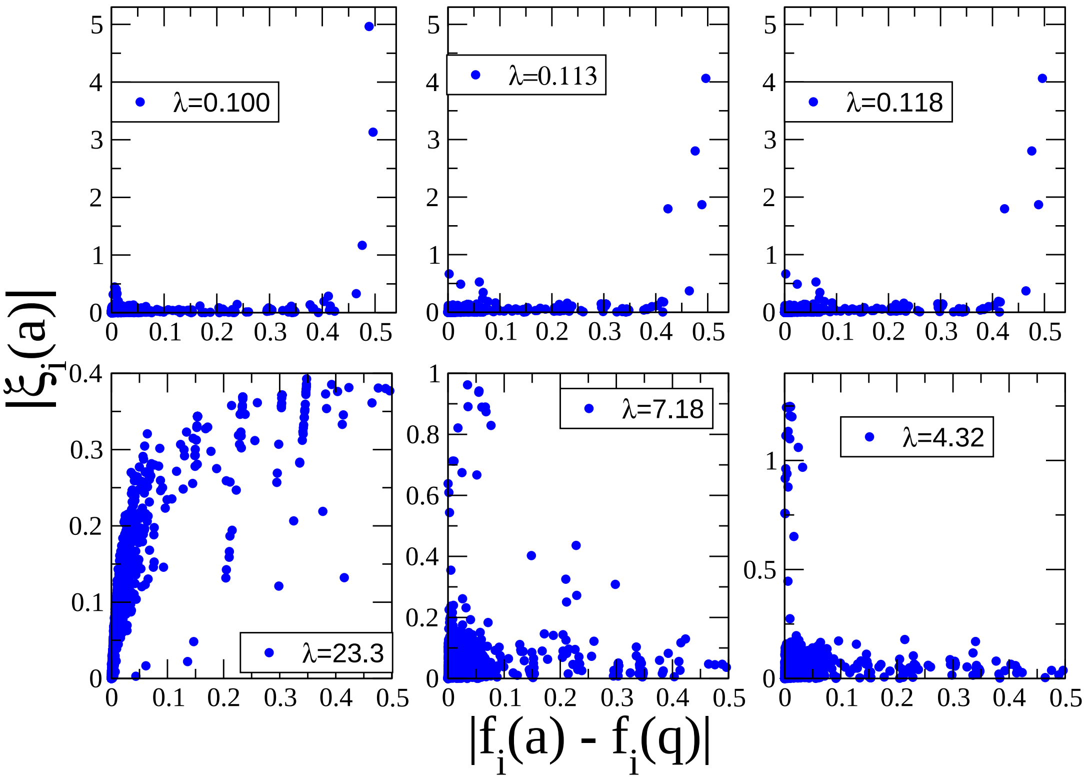

Figure S9: **Pattern components vs. residues frequencies for PF00072.** The strong correlation between large $\xi_i(a)$ and large $f_i(a)$ shows that the top attractive patterns correspond to most conserved sites.

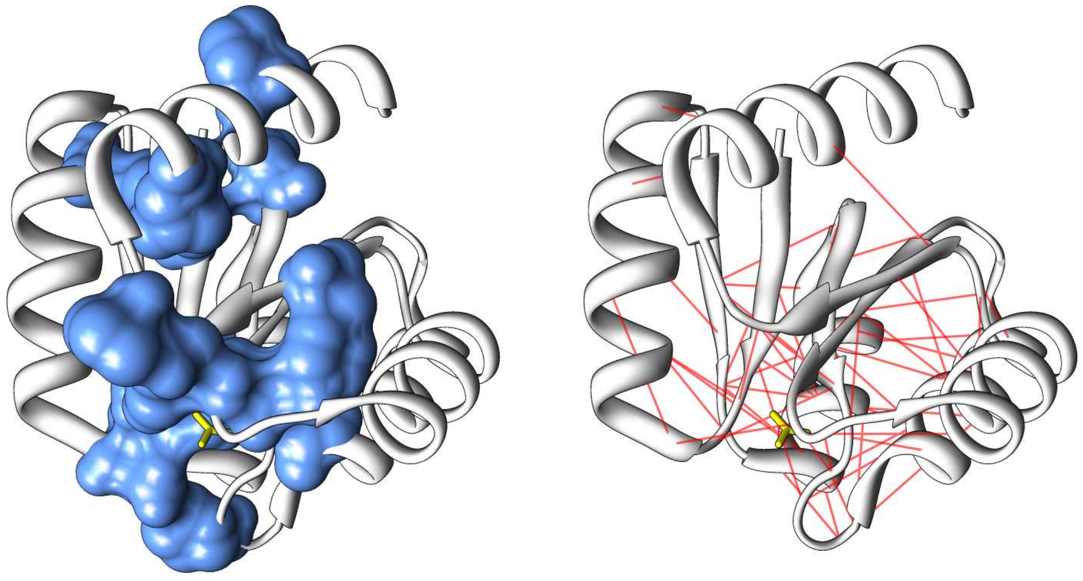

Figure S10: **Conservation and contacts on the 3D fold for PF00072**

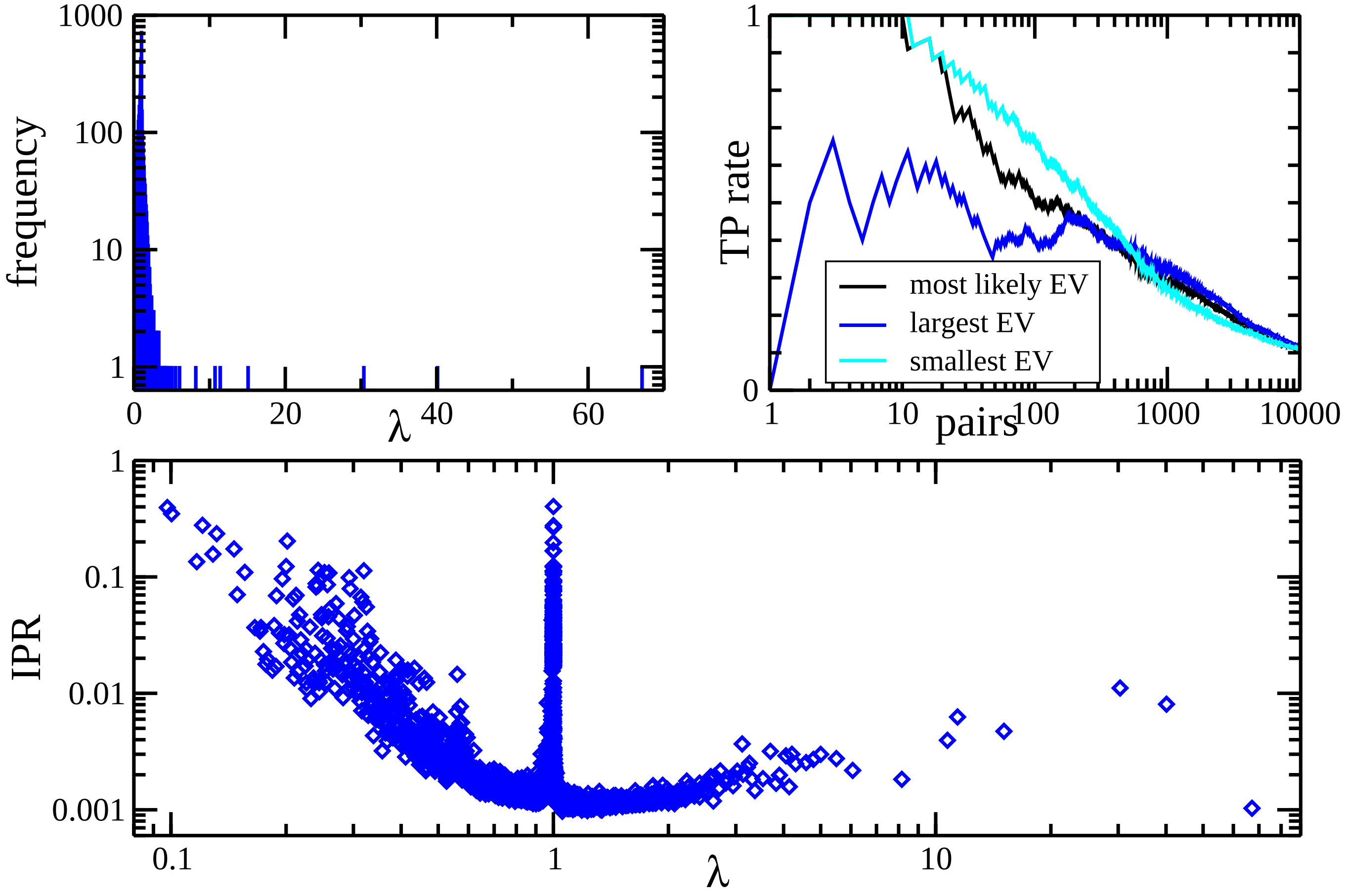

Figure S11: **Localization and contact prediction for PF00071.** *(Upper left)* The spectral density as a function of the eigenvalues λ, note the existence of few very large eigenvalues, and a pronounced peak in $\lambda = 1$. *(upper right)* TP rate for the contact prediction using the 100 most likely Hopfield patterns (black), and the patterns corresponding to the 100 smallest resp. largest eigenvalues (red resp. blue). *(lower panel)* The inverse participation ratio of the Hopfield patterns as a function of the corresponding eigenvalue λ.

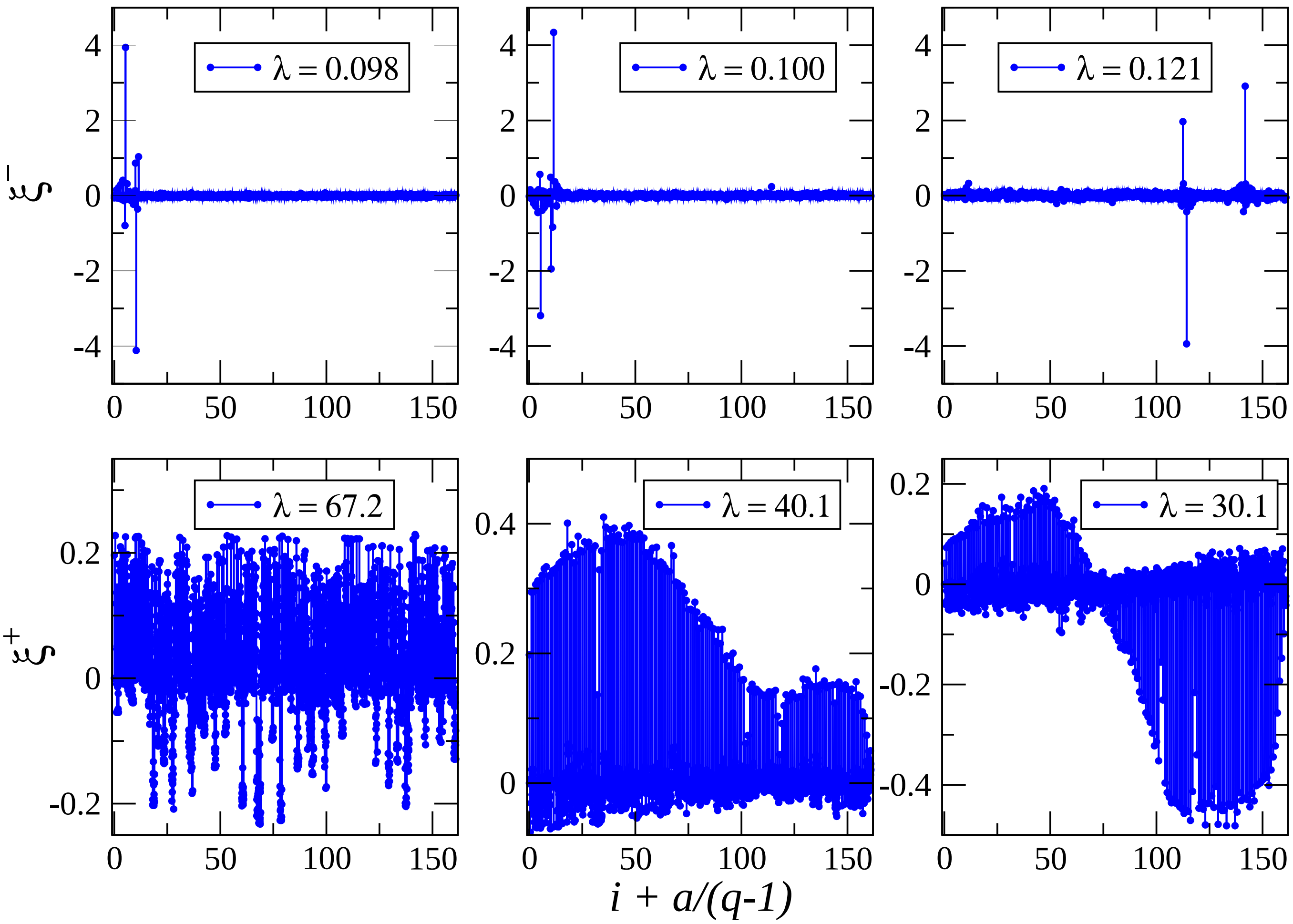

Figure S12: **Attractive and repulsive patterns for PF00071.** *(Upper panels)* The most repulsive patterns (corresponding to the first, second and fourth smallest eigenvalues) are strongly localized over pairs of sites with inverse participation ratios $0.39, 0.35, 0.28$ respectively. *(lower panels)* Shown are the most attractive patterns (corresponding to the three largest eigenvalues); the top pattern is extended with inverse participation ratios 0.001 , while the second and third patterns, with inverse participation ratios 0.008, 0.01 respectively, have essentially non-zero components over the gap symbols only concentrated on the edges of the sequence . Note the x-coordinates $i + a/(q-1)$; its integer part is the site index, i, and the fractional part multiplied by $q-1$ is the residue value, a.

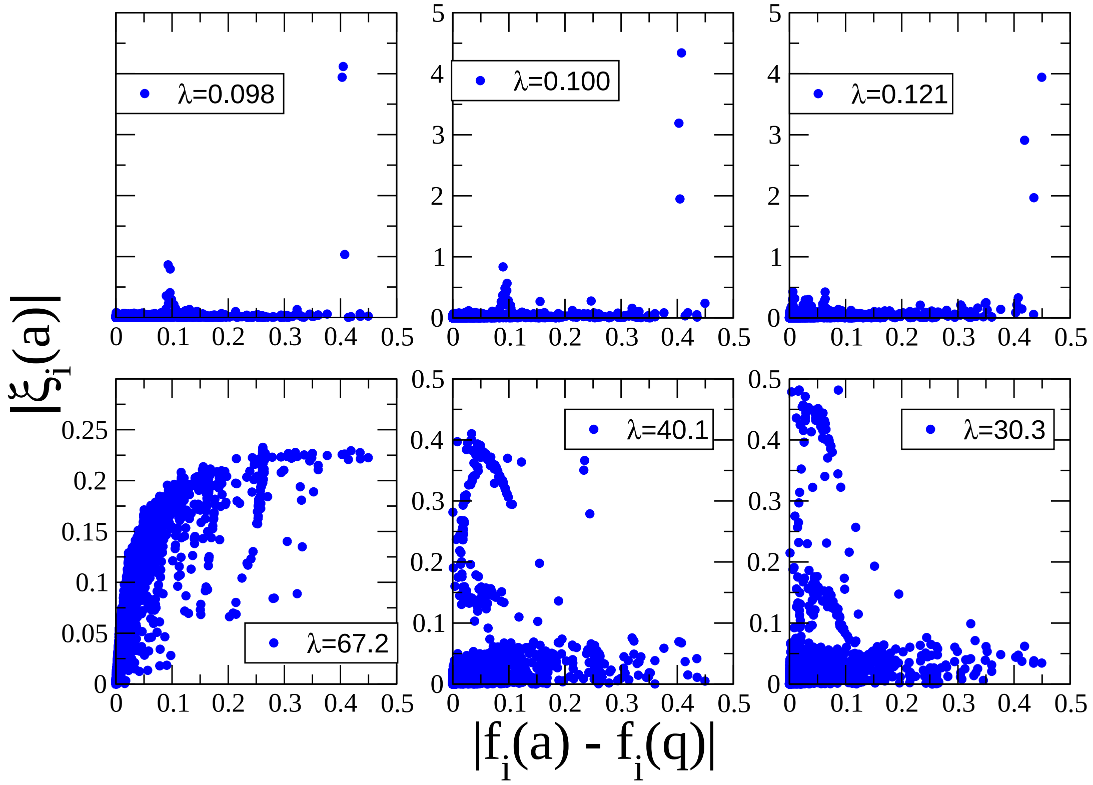

Figure S13: **Pattern components vs. residues frequencies for PF00071.**

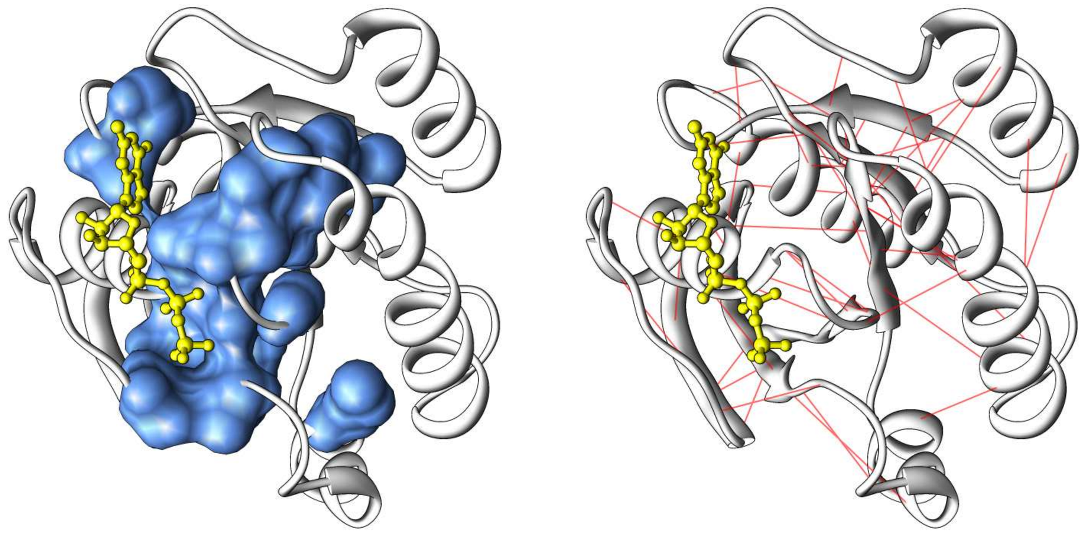

Figure S14: **Conservation and contacts on the 3D fold for PF00071**

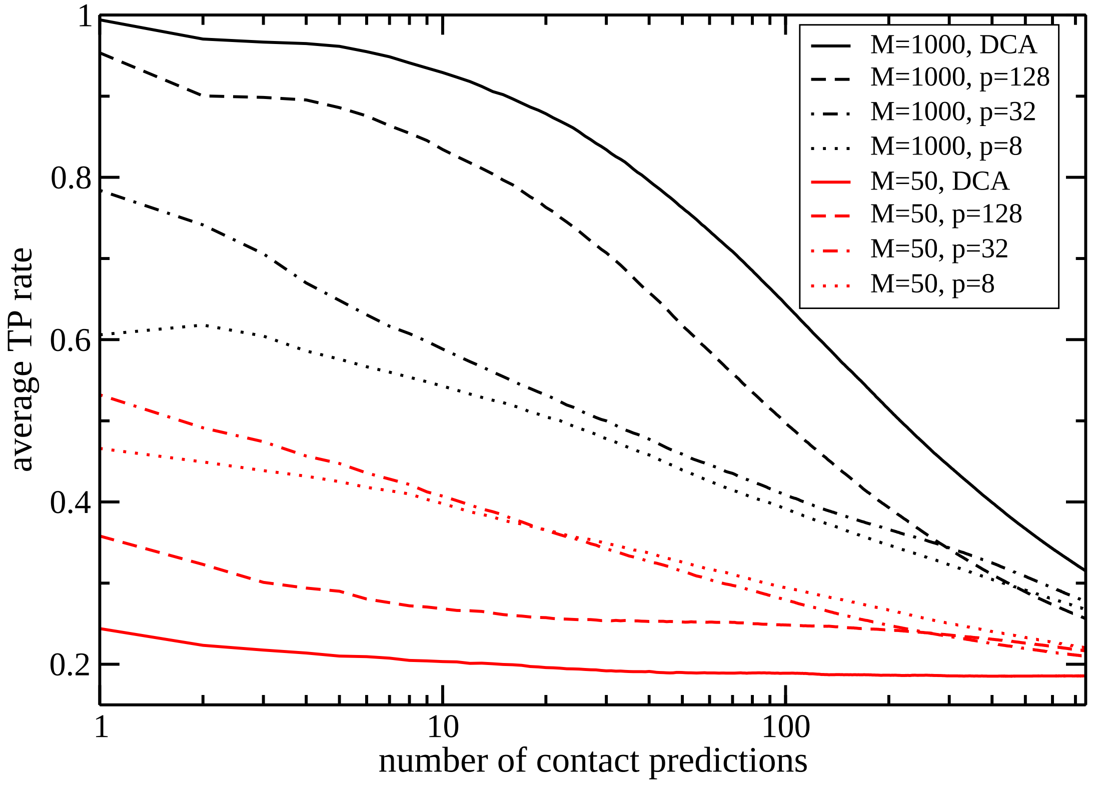

Figure S15: **Contact prediction in MSA of reduced sequence number.** TP rates are averaged over 15 protein families, for reduced MSA size of $M = 1000$ (black lines) and $M = 50$ (red lines) sequences (100 random sub-MSAs for each family and each value of M and p). Whereas for large MSAs it is optimal to use full mean-field DCA (i.e. $p = L(q-1)$), for small MSA a clear optimum in an intermediate value of p is observed, here $p = 32$. Larger MSA lead to a larger optimal p, smaller to a smaller optimal value. The actual value depends also on the family and the random realization of the sub-MSA.
